# Metabostemness: A New Cancer Hallmark

**DOI:** 10.3389/fonc.2014.00262

**Published:** 2014-09-29

**Authors:** Javier A. Menendez, Tomás Alarcón

**Affiliations:** ^1^Metabolism and Cancer Group, Translational Research Laboratory, Catalan Institute of Oncology-Girona (ICO-Girona), Girona, Spain; ^2^Girona Biomedical Research Institute (IDIBGI), Girona, Spain; ^3^Computational and Mathematical Biology Research Group, Centre de Recerca Matemàtica (CRM), Barcelona, Spain

**Keywords:** stemness, metabolism, reprograming, cancer stem cells, oncometabolites, Waddington, epigenetic landscapes

## Abstract

The acquisition of and departure from stemness in cancer tissues might not only be hardwired by genetic controllers, but also by the pivotal regulatory role of the cellular metabotype, which may act as a “starter dough” for cancer stemness traits. We have coined the term metabostemness to refer to the metabolic parameters causally controlling or functionally substituting the epitranscriptional orchestration of the genetic reprograming that redirects normal and tumor cells toward less-differentiated cancer stem cell (CSC) cellular states. Certain metabotypic alterations might operate as pivotal molecular events rendering a cell of origin susceptible to epigenetic rewiring required for the acquisition of aberrant stemness and, concurrently, of refractoriness to differentiation. The metabostemness attribute can remove, diminish, or modify the nature of molecular barriers present in Waddington’s epigenetic landscapes, thus allowing differentiated cells to more easily (re)-enter into CSC cellular macrostates. Activation of the metabostemness trait can poise cells with chromatin states competent for rapid dedifferentiation while concomitantly setting the idoneous metabolic stage for later reprograming stimuli to finish the journey from non-cancerous into tumor-initiating cells. Because only a few permitted metabotypes will be compatible with the operational properties owned by CSC cellular states, the metabostemness property provides a new framework through which to pharmacologically resolve the apparently impossible problem of discovering drugs aimed to target the molecular biology of the cancer stemness itself. The metabostemness cancer hallmark generates a shifting oncology theory that should guide a new era of metabolo-epigenetic cancer precision medicine.

## Introduction

We are accumulating ever-growing evidence that metabolism and stemness are highly intertwined processes in tumor tissues. Cancer is beginning to be understood as a disease of reprograming that appears to involve the progressive resetting of the metabolic infrastructure and metabolite levels concomitantly with changes in cellular differentiation (1–9). The modulation of metabolism and associated signaling is being increasingly implicated in the determination of cell identity during nuclear reprograming and oncogenesis. The transformation of cellular metabolism precedes changes in stemness and, therefore, metabolic reprograming appears to reflect the molecular dynamics fundamental for the rearranging and redirection of cell-fate (10–30). Moreover, we have recently learned that certain metabolites can be oncogenic themselves and, crucially, the malignant activity of these oncometabolites, i.e., small-molecule components (or enantiomers) of normal metabolism whose accumulation causes signaling dysregulation to establish a milieu that initiates and drives carcinogenesis (31–45) likely relies on their ability to epigenetically block the acquisition of differentiation markers while inducing the expression of stem cell maintenance genes. A key question, however, remains unanswered: how can metabolism and metabolites exert influence over the transcriptional factors, the chromatin structure, and the epigenetic circuits that establish and maintain the self-renewal and differentiation capacities owned by cancer stem cells (CSCs), which are suggested to drive tumor-initiation and metastatic progression?

Herein, we propose that the molecular logic behind the conversion of non-CSCs into CSCs can be better understood in terms of cellular metabotypes that operate as pathways or roadblocks by facilitating or impeding, respectively, the epitranscriptional orchestration of the genetic reprograming that drives the intrinsic and microenvironmental paths to CSC cellular states. We therefore postulate that a bona fide metabolo-epigenetic reprograming of stemness exists in pre-malignant and cancer tissues, a new cancer trait to which we have coined the name “metabostemness.”

## Metabolism: Emerging as a Cancer Hallmark

When Hanahan and Weinberg revisited the hallmarks of cancer in 2011 (46), they raised the question of whether the deregulation of cellular metabolism in tumor tissues might be viewed as a core hallmark capability of cancer cells that is as fundamental as the six well-established core hallmarks formerly proposed in 2000 (47). Alternatively, the reprograming of cancer metabolism might be merely viewed as an evolutionary conserved target that is upstream programed by oncogenic gain-of-function events and the loss of tumor-suppressors (48). The latter view implies that a stereotyped pattern of cancer-associated metabolic changes including accelerated glucose transport, reduced mitochondrial oxidative phosphorylation (OXPHOS) accompanied by aerobic glycolysis and lactate production (i.e., the Warburg effect), and augmented *de novo* fatty acid biogenesis (i.e., the lipogenic phenotype), can be all induced by most common genetic alterations in the oncogenic PI3K/AKT/mTOR/HIF axis and in the tumor-suppressor p53 system (49–53). Not surprisingly, the metabolic signatures of cancer cells have been frequently perceived by traditional biochemists as indirect, secondary phenomena that are merely required to support oncogene-directed anabolic proliferation and survival. Instead of adopting the challenging notion that tumor cells might essentially exhibit increased autonomy in maintaining an anabolic phenotype because proto-oncogenes and tumor-suppressors originated through evolution as components of metabolic regulation, Hanahan and Weinberg rather considered cluster analyses showing that several cancer-driving mutations converge on metabolic pathways. Subsequently, they designed cancer metabolic reprograming as an “emerging hallmark” to highlight the unresolved issues surrounding its functional independence from the bona fide cancer hallmarks (46, 47).

## Stemness: A forgotten Core Cancer Capability

Several researchers have advocated incorporating the two key properties of stem cells, i.e., the ability to proliferate without lineage commitment (i.e., self-renewal), and the capacity to differentiate into one or more specialized cell types (i.e., pluripotency), as a new-dimensional hallmark of cancer (54–58). The role of stemness as a cancer attribute was originally identified from the analysis of the outcomes of high-throughput gene expression datasets revealing that biologically aggressive, poorly differentiated tumors display transcriptional profiles characterized by the overrepresentation of gene signatures usually enriched in embryonic stem cells (ESCs) (59–63). Some carcinomas appear to hijack the stemness transcriptional factors’ machinery to support tumor-initiation, aberrant proliferation, and metastasis; accordingly, the activation of reprograming-like dedifferentiation mechanisms driven by master regulators of self-renewal and pluripotency (e.g., Sox2, Oct4, and Lin28) has been repeatedly shown to generate cell populations enriched with CSC-like cells that possess tumor-initiation and colonization capacities (64–72). However, the pioneer suggestion by Bond et al. (73) almost 20 years ago that the apparent dedifferentiation accompanying malignant progression can play a causal rather than passive role in the critical tumors-behavior-switch from well-differentiated to highly aggressive forms has been commonly forgotten. Most cancer researchers have adopted an alternative view, in which tumors adhere to essentially irreversible top-down hierarchies of CSC-driven cellular differentiation that caricature those occurring in normal tissues. As for metabolic reprograming, the stemness-related loss of differentiation, one fundamental characteristic of most tumor tissues, was not considered a distinct hallmark in the framework provided by Hanahan and Weinberg in 2011.

## Stemness in Cancer Tissues: What is the Origin of Cancer Stem Cells?

Carcinogenesis involves the accumulation of numerous mutational events over long periods of time. In tumors that originate from tissues with high cellular turnover, only adult stem cells (ASCs), with their innate self-renewal capacity, can remain in the tissue long enough to accumulate the number of oncogenic alterations that are necessary to support a complete malignant transformation. This has led to the hypothesis that tumor-initiation and progression are driven by CSCs, commonly defined as the fraction of tumor cells specifically endowed with self-renewal and tumor-seeding potential and the ability to spawn non-CSC progeny (74–76). Not surprisingly, ASCs have been commonly hypothesized to represent the cells of origin in most tumors because they can be directly targeted with primary transforming events; more committed progenitors can also similarly gain the ability of self-renewal and function as CSCs through oncogenic transformation. In this hierarchical organization with rare, self-renewing CSCs residing at the top, the disorganized tumoral mass should be merely viewed as an aberrant version of the ASC-driven mechanisms that govern the corresponding tissue’s normal development (77–79). However, while this is the case for cancers with a stem cell origin such as hematopoietic malignancies, for those with non-stem cell origin including liver, breast, lung, pancreatic, and prostate cancers, although CSCs obviously exhibit stem cell properties, they do not necessarily originate from the direct transformation of normal tissue stem cells or progenitor cells.

We now know that non-cancerous and differentiated cancerous cells possess enough plasticity to aberrantly reprogram and acquire bona fide CSC properties. Indeed, the inherent aggressiveness of carcinomas appears to derive not from the pre-existing content of CSCs, but rather from the intrinsic proclivity of a given tumor tissue to generate new CSC from non-CSC cell populations (80–90). Such plasticity potential of non-CSCs to acquire a CSC cellular state depending on their epigenetic/transcriptional signature and in their interpretation of multiple microenvironmental signals (e.g., hypoxia, starvation, inflammation, and therapeutics) is fully absent in the conventional depiction of the one-way stem/progenitor cell hierarchy and is revolutionary changing our current perception of the CSCs’ biology. By understanding cancer as a disease of differentiation, CSCs can be generated at any time during cancer progression so long as appropriate oncogenic lesions that can enable epigenetic reprograming to a stem-like cellular state are present or, alternatively, tumor-suppressors are hindered. Because CSCs are emergent, dynamic cellular states, i.e., CSCs are also made and not just born, multiple independently derived and molecularly distinct CSC cell populations may evolve depending on the likelihood of reprograming phenomena within tumors. The resulting heterogeneity manifests as diverse clones of CSC that vary in terms of their dormancy, proliferative, biomarkers, metastatic, and/or chemo-sensitivity profiles. Indeed, the molecular heterogeneity and stochasticity of gene expression in cancer tissues can drive a continuum of cancer cell states to rapidly shape cancer’s evolution through a greater probability of entering CSC cellular states.

## Cancer Stem Cells Reprograming: A New Paradigm for Understanding Tumors’ Social Structure

Cancer stem cell reprograming is a molecular process able to establish the bidirectional control of tumors’ epigenetic hierarchy. On the one hand, cancer genetic alterations can reset the epigenetic and transcriptional status of an initially healthy cell to establish a newly acquired, pathological differentiation program of aberrant stemness (i.e., a CSC-like cellular state) that ultimately leads to cancer development. On the other hand, differentiated tumor cells can dynamically alter their transcriptional and epigenetic circuits to acquire a less-differentiated CSC cellular state. Differentiated (normal and tumor) cells and CSCs are therefore distinct cellular states that could convert each other to achieve a balanced equilibrium within heterogeneous cancer cell populations. Crucially, the reprograming-differentiation cancer model easily explains many of the apparently paradoxical aspects of the CSC-related tumor biology: (1) the apparently contradictory reconciliation of the rarity (of CSC number) with robustness (of CSC properties) in some tumors; (2) the challenging application of hierarchical models to some tumors such as metastatic melanoma [i.e., an extreme example of stem cell reprograming in which certain epigenetic makeups endow almost the entire tumor cell population with the easiest ability to acquire CSC qualities; (91)]; (3) the lack of bona fide CSC markers enabling the general identification of stemness across different cancer types and even during the natural history of a given tumor type (61, 92–94); (4) the occurrence of unique stem-like states due to the continuous evolution and adaptation to new constraints [e.g., the conversion of tumor cells into functional vascular endothelial cells that resist antiangiogenic therapy or the transient assumption by individual cells of a reversible drug-tolerant state to protect the cancer cell population from eradication due to potentially lethal exposures; (95, 96)]; and (5) the accumulation of CSCs following treatment with therapeutics [i.e., cancer therapies do not necessarily enrich cancer tissues with pre-existing, treatment-refractory CSCs, as an accelerated production of *de novo* CSC cellular states from the residual cancer cells may easily repopulate their ranks while the older CSCs die; (97)]. The reprograming-dedifferentiation cancer model illuminates the fact the “hierarchy” within tumors’ social structure is not rigid because self-renewal and differentiation are acquired traits. Importantly, CSC reprograming does not exclude the pivotal role of non-strictly genetic factors (e.g., metabolites and microenvironment), which can significantly impact the conversion probability between non-CSCs’ and CSCs’ cell stages.

## Cancer Metabostemness: A Conceptual Description

A key peculiarity of the abovementioned model of stem cell reprograming is that the dysregulation of specific signaling pathways, rather than the type of the cell of origin, dictates the emergence and phenotype of CSCs in a given tissue. If any differentiated cell can be reprogramed to an induced pluripotent state through the right combination of transcription factors, then, following the same line of reasoning and in theory, non-CSC cells could similarly dedifferentiate to a CSC cellular state given that an appropriate stemness transcription factor is strongly activated. Moreover, by balancing counteracting differentiation forces (98), reprograming to a CSC functional state might be also achieved through the establishment of a fine-tuned equilibrium that might not require of the traditionally considered master regulators of stemness. However, by following the numerous parallels between the process of reprogramming differentiated cells-to-induced pluripotent stem cells (iPSCs) and differentiated cells-to-CSCs, when considering the slow kinetics and efficiencies of iPSCs generation (99) it might tempting to suggest that not all of the normal or cancerous cells would possess the equivalent ability *ab initio* to permit their successful reprograming to CSC cellular states. Whereas certain populations are seemingly refractory to reprograming, we now know that the ability to generate iPSCs is intrinsic to any cell given sufficient time and the appropriate reprograming push. Thus, additional expression of the so-called Yamanaka factors, the use of additional stemness transcription factors, the use of chemicals, or the direct modification of critical epigenetic components can greatly enhance (even to efficiencies nearing 100%) and accelerate reprograming to iPSCs (100–104). Ever-growing iPSCs-based findings showing that metabolic reprograming phenomena might be essential for transcription factor-induced stemness have further confirmed that the elaborate regulation of key master metabolic switches seems to contribute to the metabolic changes that take place in the transition between a differentiated cell and a stem cell, to the maintenance of the stemness properties in stem cell cellular states, and to the exit from the pluripotent state to become primed for differentiation (10–30, 105). In this scenario, we recently reasoned that the dedifferentiation of somatic cells into iPSCs as well as the *de novo* generation of CSC cellular states from non-CSCs may represent mechanistically related, metabolically dependent reprograming phenomena in which epigenetic remodeling, the activation of genes related to the establishment and maintenance of stemness, and/or the scavenging of fate determinants related to cell differentiation might be co-opted in the absence of functional tumor-suppressing mechanisms. In other words, the metabolic infrastructure and functioning might operate as the key molecular constraint controlling the kinetics of stemness reprograming for the optimal routing of non-CSC to CSC-like cellular states during cancer genesis and progression.

**Figure 1:**
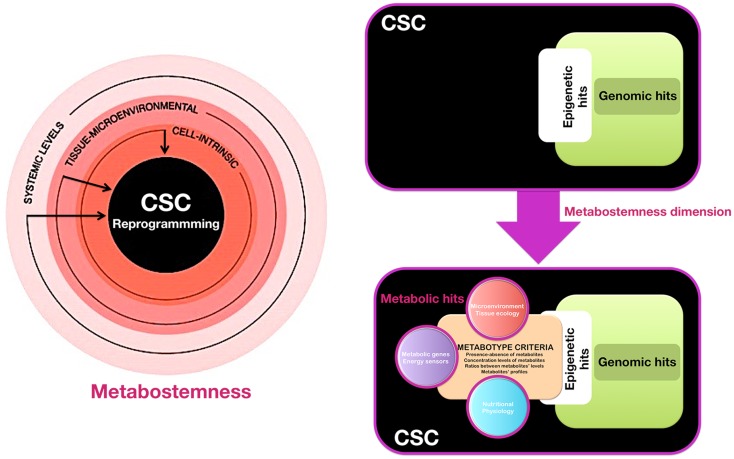
**Metabostemness: a new-dimensional cancer hallmark**. We have recently hypothesized that CSC-driven malignant progression might be envisioned as an evolving spatio-temporal heterogeneous structure that might not be driven solely by irreversible genomic hits but also by metabolic means; thus, the acquisition of, and departure from, stemness in cancer tissues might be governed not only by transcriptional and epigenetic controllers but also by the pivotal regulatory role of metabolic reprograming in cell-fate decisions. In this scenario, we propose the actual existence of a new phenomic cancer hallmark to which we have coined the term “metabostemness.” Metabostemness refers to the metabolic parameters at the cell-intrinsic, tissue-microenvironmental, and systemic levels that enable the unique functional properties owned by the CSC cellular states. A metabotype-based infrastructure and functioning of CSC can therefore operate as a supra-genetic dimension controlling over or functionally substituting the epitranscriptional orchestration of the genetic reprograming that redirects normal and non-CSC tumor cells toward less-differentiated CSC cellular states.

We propose that the acquisition of and departure from stemness in pre-malignant and cancer tissues might not be governed exclusively by genetic and epigenetic controllers, but also by the pivotal regulatory role of the cellular metabotype, which may act as “starter dough” for cancer stemness traits. When viewing cancer stemness as a flexible quality that might be gained and lost in a metabolic-dependent manner, the cellular metabotype then operates as a supra-genetic dimension guiding the ability of epigenetic and transcriptional circuitries to redirect normal and non-CSC tumor cells toward a CSC-like cellular state. Certain metabotypic shifts might function as very early molecular events that render a (normal or cancerous) differentiated cell more susceptible to transcriptional and epigenetic rewiring required for the acquisition of aberrant stemness and, concurrently, of refractoriness not only to apoptosis, but also to differentiation. Subsequent hits, occurring in variable orders, combinations, and/or intensities can then confer the definitive quality of cancer stemness and dictate the dynamic cellular hierarchy within a tumor tissue. We have therefore coined the term “metabostemness” to refer to the metabolic parameters causally controlling or functionally substituting the epitranscriptional orchestration of the genetic reprograming that redirects normal and non-CSC tumor cells toward less-differentiated CSC cellular states (Figure 1). As such, the metabostemness trait can be understood as the physiological glue that metabolically connects all the omic layers with a self-autonomous CSC cellular quality; operatively, metabostemness is a systeomic function of observable metabolic phenotypes (i.e., the CSC metabolophenome) that predate systems biology and its sub-disciplines (i.e., genomics, transcriptomics, proteomics, and metabolomics) at the level of CSC cellular states. From a holistic perspective, the metabostemness hallmark comprises the intrinsically, microenvironmental, and physiologically determined metabolic parameters that enable the self-renewal and differentiation capacities owned by the CSC cellular states in tumor tissues (Figure 1). Four main features can conceptually define the metabostemness property in cancer tissues:

(1) the cellular metabotype determines the global success of the epitranscriptional reprograming that redirects normal and non-CSC tumor cells toward less-differentiated CSC cellular states;
(2) the cellular metabotype imposes the “permitted” and “protected” cellular modes that allow or prevent, respectively, the completion of the molecular journey from non-CSC to CSC cellular states;
(3) the closer a cellular metabotype is to that of a CSC, the higher its reprograming capacity for acquiring a CSC cellular state; and
(4) metabolic interventions can reprogram cellular metabotypes in a manner that successfully impedes the aberrant acquisition and functioning of stemness in cancer tissues.

## Cancer Metabostemness: An Operational Delineation

Transcription factors are commonly viewed as the key intrinsic regulators of cell-fate, i.e., the cell-fate choice exclusively involves modulating networks of transcription factors. It is also generally accepted that a given cellular type including that of stem cells must not be excessively sensitive to random, or unpredictable, small fluctuations in the levels of specific signals. This indispensable requirement to withstand modulating factors that may perturb the gene regulatory network (GRN) architecture – i.e., the gene–gene relationships, their directionality (“who controls whom”), their interaction modalities (inhibition versus stimulation), and the modes of cooperation – defines a cell type in a timely manner and strongly limits the number of solutions or “cellular states” for a given genetic system including those of cancer tissues (106–108). The evolving dynamics of molecular connections between the genes and gene products that can interact with each other within a cell, including the underlying regulatory logic that govern these interactions, must therefore provide robustness with respect to varying extrinsic signals and intrinsic factors, i.e., the noise. Such stability of the cell state is obviously required in stem cells to support the self-renewal and maintenance of an uncommitted state, but must also afford certain flexibility in the choice of cell-fate to permit the diversification and differentiation of cell types in response to intrinsic cues or extrinsic signals.

During normal development, cells make very precise transitions between network states of gene expression patterns, which must be stable and irreversible in terminally differentiated cells, at least under homeostatic or physiological conditions. Conversely, the potential for reverse differentiated-to-stem cell state conversions has been revolutionarily exemplified by the nuclear reprograming of somatic cells to a pluripotent stem cell state driven by a small number of stemness transcription factors. Crucially, a very similar consideration of cell states transitions apply to pathological states such as cancer, where the misexpression of transcriptional regulators can reset the status of an initially healthy cell to establish a newly acquired, pathological differentiation program of aberrant stemness that ultimately leads to cancer development. Considering the above-depicted scenario, it is not surprising that the major challenge that is being faced by regulatory biology in the postgenomic era of understanding cancer diseases is to map the core transcription factors’ networks associated with different cancer cell types, especially the underlying regulatory logic that governs their behavior as differentiated versus CSC cellular states. The accurate delineation of such a map will provide crucial insights into the rules that define cancer cellular states (i.e., cancer heterogeneity) and how transitions between cancer cellular states are achieved, thus providing unforeseen therapeutic solutions to the apparently irresoluble problem of targeting cancer dynamical models such as the model of reversibility, or cancer cell reprograming, in which heterogeneous populations of CSC can arise by reversion of more differentiated cancer cells. Indeed, multiple different CSC populations have been described for a given cancer type, despite presenting different gene and protein expression signatures, thus leading to the currently accepted view that the characterization of CSCs can no longer be based on marker expression, but instead at their functional level, strongly supporting the model of reversibility, or CSC reprograming, in which the populations of CSCs arise by reversion of more differentiated cells (85, 109–113).

As for bona fide pluripotency, CSCs should be viewed as functional states rather than discrete cellular entities characterized by well-defined and static gene networks, thus highlighting cancer stemness as a statistical property resembling a macrostate in statistical physics (108, 114, 115). If these macrostate entities of functional CSCs are correct, the gene expression signature for a CSC in a given tumor tissue may be dynamic or non-unique, which can create a challenge when trying to unambiguously establish mathematical frameworks describing CSC reprograming phenomena. We propose that incorporating metabolism and metabolites into the intrinsic variability of the CSCs’ epigenetic and genetic signatures can significantly reduce the high-dimensional problem of understanding reprograming dynamics both at the single-cell level and the population level. But how can we model the incorporation of the metabolism and metabolites into the epigenetic and genetic signatures accounting for CSC variability during the generation, maintenance, and evolution of CSC cellular states in cancer tissues? To definitely consider the process of reprograming to CSC into a rigorous, quantifiable theory, the establishment of a mathematical framework able to accurately mapping the landscape pertaining the transition between non-CSC and CSC cell states needs to incorporate a never before considered metabolic dimension.

### Dynamic perspective by developing probabilistic descriptions of cell states

**Figure 2:**
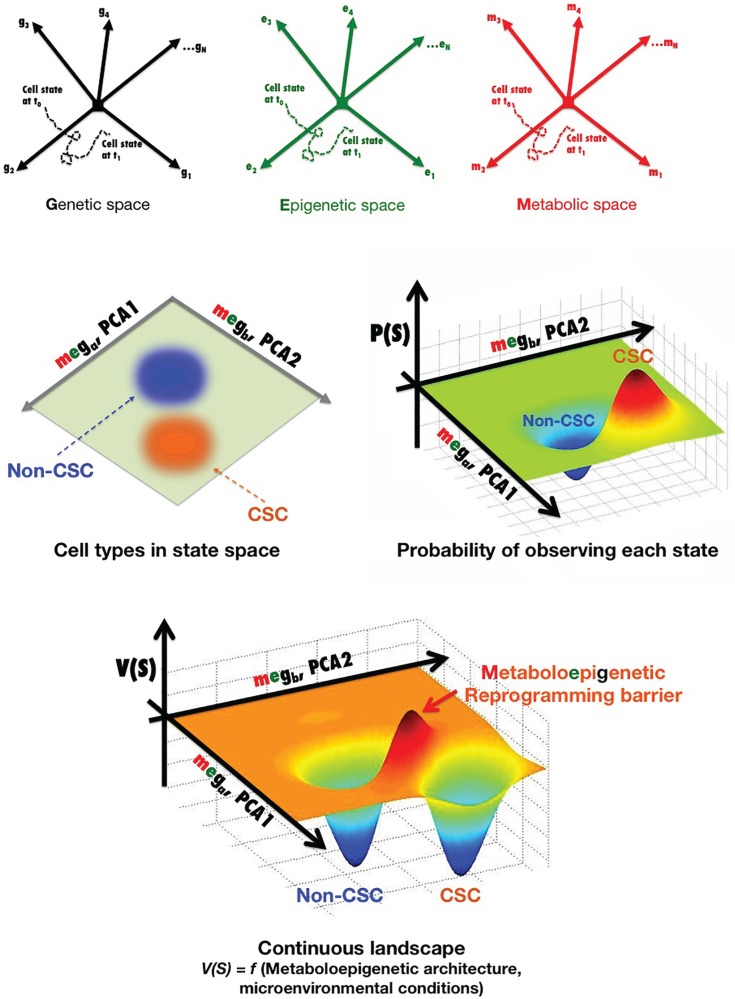
**Metabostemness: developing probabilistic descriptions of non-CSC and CSC cell states**. (See text for a more detailed explanation).

Cancer cell states (e.g., non-CSC versus CSC) can be parameterized as vectors of molecular characteristics, *Ŝ*, which are generally considered a set of gene expression levels (*Ŝ* = [*g*_1_, *g*_2_, *g*_3_, …, *g_N_*]) (Figure 2). A cancer cell at any time *t* can exist in a point of this state space, and its state can change with time in response to particular cell-autonomous and non-cell-autonomous conditions, i.e., in a CSC reprograming scenario, *Ŝ* is a function of time *Ŝ*(*t*) as a result of noise, reprograming, or differentiation. By plotting one trajectory during a state change from *t*_0_ to *t*_1_, we can fully describe the transition between cancer cell states during this time *S*(*t*) = [*g*_1_(*t*), *g*_2_(*t*), *g*_3_(*t*), …, *g*_N_(*t*)]. For simplicity, we show a cancer cell state space generated by the level of *N* different genes, *g*_1_ to *g_N_*, where each arrow represents an axis corresponding to the expression level of that particular gene transcript. Such a cancer cell state space provides an accurate means to quantitatively organize and visualize different states of a cancer cell with a fixed genome. Indeed, often cancer cells will cluster in particular regions of the cancer cell state space, which can be viewed as stable types of cancer cells that express particular markers. Those regions where no cancer cells are found correspond to cell states that are somehow unstable for the given genome of the cancer cell in the microenvironmental conditions considered. Thus, *N* is generally reduced to two to three more manageable dimensions through statistical techniques [e.g., principal component analysis (PCA)]. Figure 2 shows a two-dimensional representation of the cancer cell state space, where each axis (*g_a_* and *g_b_* from PCA) is a linear combination of genes *g*_1_ to *g_N_* and, therefore, stable types of cancer cells exist at particular points of this graph. By quantitatively mapping the gene expression levels of a large sample of single stable cancer cells in the same space, the probability of occupying each point in this space can be plotted in a continuous fashion, and regions with high densities of spots can define observable cancer cell types. The continuous probabilistic description of cancer cell types staying at a particular point in state space can be represented as a landscape, by calculating − ln [*P* (*Ŝ*)] at each point. In this landscape, *V* (*Ŝ*) represents “energy barriers” between transitions involving any two cellular states and thus may provide a more thorough description of non-CSC-to-CSC transitions. However, we should acknowledge that while the nature of the cancer cell substates can be elucidated in terms of a landscape picture in which stable differentiated, non-CSC, and CSC cellular states are mathematically defined as “attractors” – i.e., observable cancer cell types – the architecture of these apparently stable “network states” is described exclusively in terms of genes, or at the most epigenetic, expression patterns. Indeed, when selecting a particular set of characteristics as being informative for the transition between two cell states of interest, functional cell types are almost exclusively defined based on levels of gene expression changes on the timescale of days to weeks. In this probabilistic framework, the genetic background defines the abovementioned architecture of the cell state space, meaning that particular gene–gene relationships, interaction modalities, and integrating transfer functions (i.e., the GRN) are largely “hardwired” by the genome. A more detailed characterization not only of gene expression, but also of biochemical noise in signaling processes (e.g., binding of epigenetic modifiers to particular genome loci, inherent stochastic nature of molecular binding events, secondary messengers, and variable cell–cell contact) and of the epigenetic state (e.g., levels of epigenetic methylation or acetylation marks on DNA or chromatin) is expected to provide more insight into the missing key parameters and required transcriptional changes controlling the kinetics of CSC reprograming. In this regard, we propose that the regulatory network architecture of a cancer landscape comprises not only the genes and proteins that can interact within a cancer cell, but also the cellular metabotype, which can necessarily and sufficiently characterize different functional cell states within heterogeneous cancer cell populations through different types and/or levels of certain metabolites.

**Figure 3:**
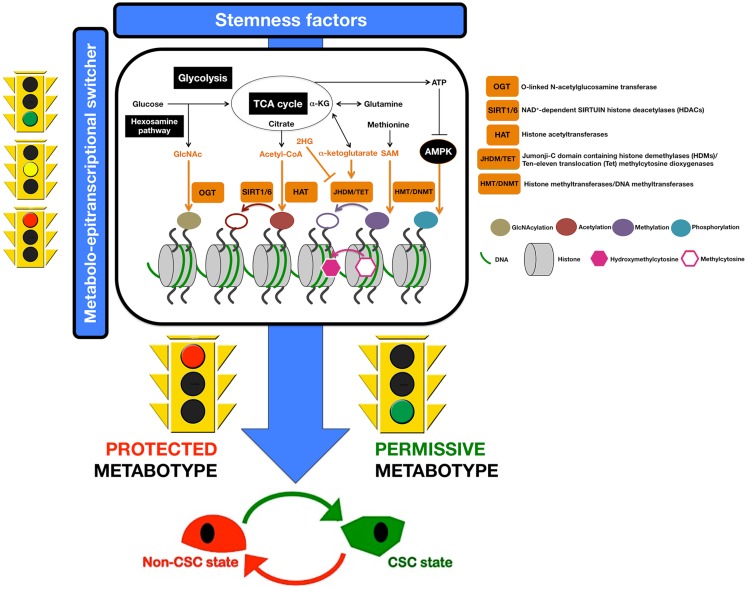
**Metabostemness: a metabolo-epitranscriptional switcher**. A highly active crosstalk between metabolism and epigenetics allows the causal integration of metabolism and metabolites with genetic programs to coordinately regulate CSC function and fate. Nutrient levels and cell metabolism will significantly affect levels of the metabolites, which are required substrates of chromatin-modifying enzymes that use them to modify both histones and DNA (116). A minor portion of the lucose that enters the glycolytic pathway is branched to hexosamine biosynthetic pathway to produce GlcNAc, a substrate for histone GlcNAcylation by the OGT enzyme. It is well known that the flux through glycolysis determines the NAD^+^/NADH ratio, a crucial metabolic parameter for the activities of sirtuin histone deacetylases. Citrate and α-ketoglutarate (α-KG) are intermediates of the TCA cycle that can be exported out of mitochondria; cytosolic citrate is converted to acetyl-CoA that is employed as a donor for HAT-mediated histone acetylation. JHDM and TET use α-KG as cofactor for histone and DNA demethylation reactions, respectively. S-adenosylmethionine (SAM), which is synthesized from essential amino acid methionine, is a donor for DNA methylation via HMT and DNMT. A low ATP/AMP ratio indicative of metabolic stress can activate AMPK, which can translocate to chromatin and phosphorylate histones. In this scenario, we propose that certain metabotypes might operate as pivotal molecular events rendering a non-CSC cell susceptible to epigenetic and transcriptional rewiring required for the acquisition of aberrant stemness and, concurrently, of refractoriness not only to apoptosis but also to differentiation. Only certain cell metabotypes would be compatible with the operational properties exclusively owned by CSC cellular states; conversely, certain metabotypes would impose cellular modes protected against CSC reprograming. In other words, the molecular logic behind the conversion of non-CSC into CSC can be better understood in terms of cellular metabotypes that operate as pathways (“permissive”) or roadblocks (“protected”) by facilitating or impeding, respectively, the transcriptional events and signal transduction pathways that lastly coordinate the intrinsic and microenvironmental paths to CSC cellular states.

But how do metabolism and metabolites hierarchically integrate with genetic expression programs to coordinately regulate CSC function and fate? Because DNA transcription is regulated by chromatin organization, the occurrence of metabolic inputs into epigenetic modifications of chromatin and transcription should be viewed as the molecular bridge that links metabolism to epigenetics and gene expression during non-CSC to CSC transitions. We are accumulating ever-growing evidence suggesting that, beyond the classically delineated transcriptional output of growth factor/hormonal signaling pathways, a variety of metabolic signals can play also critical roles in determining chromatin structure, thus directly linking metabolic perturbations to the dysregulation of cellular differentiation (116–118) (Figure 3). Indeed, CSC metabolism is linked to epigenetics and gene expression in a multifaceted and bidirectional manner:

– Metabolic fluxes can be controlled by metabolic enzymes that are directly regulated by stemness transcription factors such as c-Myc, which can act both locally and globally on chromatin to exert wide-ranging effects on the biology of stem and tumor cells (119–122).
– Epigenetic alterations (e.g., demethylation or methylation of gene promoter regions, protein acetylation) can contribute to the deregulated expression of key enzymes involved in cell metabolism. Thus, the reversible acetylation of histones and non-histone proteins has been shown to affect cell metabolism, and glycolytic versus OXPHOS pathway gene expression and DNA methylation patterns change during reprograming to stemness. The rate-limiting glycolytic enzyme Hexokinase II (HK2) and the key enzyme of gluconeogenesis fructose-1,6-biphosphatase (FBP1), with opposing roles in glycolysis, have recently been identified as epigenetically regulated by promoter demethylation and methylation, respectively (123–126). PKM2, which catalyzes the conversion of phosphoenol pyruvate to pyruvate, is targeted for degradation in a glucose-dependent manner by acetylation at lysine K305; the epigenetic silencing of FBP1, which catalyzes the energy-consuming conversion of fructose-1,6-biphosphate to fructose-6-phosphate, is employed by CSC as a mechanism of glucose flux maintenance via glycolysis and other associated biosynthetic pathways (127). The tumor tissues’ extra requirements of glucose, an essential nutrient for CSC that, when present in the culture environment, significantly increases the percentage of CSC-like cells in the overall cancer cell population (128), can be achieved via the epigenetic silencing of DERL3, the gene responsible for degrading the glucose transporter SLC2A1 [glucose transporter 1 (GLUT1)] (129). Moreover, posttranscriptional modifications of p53, the key connector of reprograming to pluripotency and tumorigenesis (130–136), may affect cell metabolism through downstream targets such as TIGAR (TP53-induced glycolysis and apoptosis regulator) and interaction with PGC-1α [peroxisomal proliferator-activated receptor (PPAR) gamma coactivator 1] (137–142).
– Epigenetic modifications of DNA and histones by methylation and acetylation reactions require cofactors that are derived from various metabolic pathways including glycolysis, fatty acid oxidation, tricarboxylic acid (TCA) cycle, and OXPHOS (Figure 3). Among these cofactors, we can mention the following: (a) S-adenosyl-L-methionine (SAM), which is a cofactor for methylation reactions by DNA- and histone-methyltransferases (DNMTs and HMTs) (143–145); (b) Flavin adenine dinucleotide (FAD), which is a cofactor for lysine specific demethylase 1 (LSD1) (144, 146); (c) α-ketoglutarate, which is an electron donor and cofactor for the α-KG/Fe^2+^-dependent dioxygenases Jumonji-C domain (JmjC) histone demethylases (HDMs) and ten–eleven translocation (TET) proteins, respectively (143, 144); (d) Nicotinamide adenine dinucleotide (NAD), which is a cofactor for the sirtuins family of histone deacetylases (HDACs) and poly(ADP-ribose)polymerase (PARP) (143, 147–151); and (e) Acetyl Coenzyme A, which is not only an important precursor for the *de novo* biogenesis of fatty acids, but also an essential cofactor for histone acetyl transferases (HATs) and the acetylation of non-histone proteins involved in cell metabolism (145, 152–155). Because alterations in the supply of these cofactors may affect DNA methylation, alter chromatin structure, and change posttranslational modifications of non-histone proteins that influence the regulation of gene expression reprograming to stemness, and because cofactors and modifying enzymes are always present at some level, the current challenge is to understand how localized fluctuations in levels of metabolites control chromatin modifiers in space and time to operate as mechanisms of specificity that prevent all genes from being regulated synchronously. Enzyme recruitment with DNA-binding factors, local depletion or excess cofactors, or the modification of spatial and temporal sublocalization of enzymes within the nucleus might likely account for a hierarchy of target chromatin regions and associated genes (116, 156), thus translating CSC metabotypes into CSC methylation maps.
– Beyond the more general effects on epigenetics brought about by changes in the availability of substrates or cofactors for enzymes that regulate chromatin structure and gene expression, mutations in metabolic enzymes have been linked to the generation of oncometabolites, which directly drives epigenetic reprograming in cancer cells. Ever-growing *in vitro* and *in vivo* studies have provided strong biochemical, cell biological, and genetic evidence that oncometabolites can drive tumorigenesis and tumor maintenance by regulating histone and DNA modifications and engaging a metabolic block in cellular differentiation. This is the case of the currently recognized oncometabolites R(-)-2-hydroxyglutarate (2-HG), fumarate, and succinate, which accumulate due to defects in the TCA cycle enzymes isocitrate dehydrogenases (IDHs), fumarate hydratase (FH), and succinate dehydrogenase (SDH), respectively. Defects in these TCA cycle enzymes caused inherited benign or malignant tumors by altering DNA and histone modifications to cause widespread transcriptional dysregulation (31–45). Indeed, the identification of cancer-associated mutations in metabolic enzymes has provided the strongest support ever for the argument that the metabolism rewiring observed in cancer plays a significant role in cancer development. Crucially, by highlighting the similarities in the nuclear reprograming pathways that are involved in the generation of iPSCs and of the tumor-initiating action of CSC-like cells, it has recently been suggested that a stemness-related hallmark of cancers may be mutations or expression changes in metabolic genes that are implicated in the regulation of DNA methylation plasticity such as IDHs (7). Lu and Thompson (116) originally suggested that, during progenitor cell differentiation, the inhibition of the HDM JHDM by the oncometabolite 2-HG, which is aberrantly synthesized by the neomorphic mutations of IDH enzymes, causes defective histone demethylation and blocks the accessibility of differentiation-related genes. The 2-HG-driven inhibition of JHDM as well as that of the TET family of DNA demethylases, which operate as failsafe mechanisms to protect promoters from aberrant DNA methylation, should lead to progressive DNA hypermethylation and permanently “lock” differentiation-related genes in a silent state; the resulting differentiation arrest might facilitate cancer development through the accumulation of undifferentiated cells capable of self-renewal. Goding et al. (7) now propose that the cell of origin dictates the metabolo-epigenetic relationship between reprograming and cancer. As originally proposed by Lu and Thompson (116), gain-of-functions IDH mutations can lead to global hypermethylation, preventing the demethylation of genes that are implicated in differentiation, and consequently promoting an increase in the number of stem cells that may be targetable by oncogenic mutations in cancers with a stem cell of origin, such as hematopoietic malignancies. For cancers with a non-stem cell origin including liver, breast, lung, pancreatic, and prostate cancers, in which mutations on metabolic genes are not widespread, Goding et al. (7) propose that an intact metabolic function of IDH would be necessary to maintain DNA methylation plasticity and a flexible epigenetic landscape, as seen during the generation of iPSCs.

Nevertheless, as metabolic abnormalities in cancer continue to be uncovered, it can be expected that new metabolites or metabolic perturbations that affect chromatin structure will be identified (53, 157). Moreover, during reprograming to a CSC cellular state, a positive feedback loop might be established between bioenergetics reprograming and the activity of stemness transcriptional factors; accordingly, there is a progressive resetting of metabolite levels that parallels the progressive changes seen in global epigenetic modifications and gene expression over prolonged times of induced pluripotency maintenance. We propose that the probabilistic description of cell states in cancer tissues can be better described by incorporating the connections between metabolism and chromatin dynamics; therefore, in order to determine how metabolism and metabolites exert influence over epigenetic and genetic reprograming circuits that establish and maintain CSCs’ self-renewal and differentiation abilities, the transition between cancer cell states should incorporate not only a set of gene expression levels, but also the metabolo-epigenetic state, i.e., *S*(*t*) = [meg_1_(*t*), meg_2_(*t*), meg_3_(*t*), …, meg*_N_*(*t*)] (Figure 2).

### Dynamic perspective using Waddingtonian landscapes

It is well-recognized that the Waddington landscape (158), a metaphoric representation of cell differentiation in which pluripotent stem cells are positioned at the top of a hill progressively losing differentiation potential while going downhill into different valleys representing irreversible differentiated cellular states, allows for the modeling of a complex network of molecular barriers governing cell-fate transitions. The complexity of the enormous number of possible intracellular factors that can mitigate or impede cellular reprograming is notably reduced into an effective energy landscape in the Waddington model, in which cell-fates are defined by stable states, known as “attractors,” and cell transitions are represented as flows from one energy state to another. In this quasi-potential Waddingtonian landscape that captures the global dynamics of high-dimensional attractor states (i.e., distinct entities and of regulatory networks representing the equilibrium solutions of how the concentrations of interacting transcripts, proteins, and other variables in a GRN evolve over time to exhibit the natural properties of cell types), the cancer cell types within a tumor tissue are therefore self-stabilizing states basically defined by their “energetic” minimum (i.e., the commitment is defined as the progression toward the minimum energy) and the shape of the potential cleft. While they are robust to small perturbations, they still allow “all-or-nothing” transitions to other attractors including those of CSCs given sufficiently high perturbations, as they are separated by “hills” that correspond to “unstable” states. The latter truly represent the “epigenetic barriers” originally proposed in the 1940s to explain cell-fate determination in contraposition to the commonly, but erroneously, usually claimed “epigenetic landscape” in which unique chromatin marks such as DNA methylation and histone acetylation/methylation control the activity of specific genes, thereby acting as the crucial modulators of cell-fate. Thus, according to a bona fide Waddingtonian model based on a rather reversed role of chromatin modification as the prima causa of lineage-specific gene expression patterns by “upstream” controlling the access of transcription factors to DNA target sites (i.e., the stemness transcription factors themselves, endowed with sequence-recognition capability, are in charge of initiating the opening of chromatin at specific sites, followed by a directional cooperation with the chromatin-modifying enzymes that they recruit to their target loci), we argue that the metabolo-epigenetic nature of the potential barriers between non-CSC and CSC cell states actually determines how metabolism and metabolites exert influence over or functionally substitute the genetic and epigenetic circuits that establish and maintain stem cell reprograming in cancer tissues. In this new scenario, the underlying regulatory logic that governs the interactions between genes and proteins should include the cellular metabotype to better define the actual “metabolo-epitranscriptional regulatory network (MGRN)” architecture of cancer tissues.

A glossary of terms might facilitate the understanding of the metabolo-epigenetic landscapes in cancer tissues. The term “metabotype” refers to a cellular phenotype characterized by different metabolites’ levels that can be described by means of four variability criteria: (a) the presence–absence of metabolites; (b) the concentration levels of metabolites; (c) the relative levels or ratios between metabolites; and (d) metabolic profiles. The term “MGRN” comprises the epigenetic state, genes transcripts, proteins, and metabolites that can interact with each other within a cell, including the underlying regulatory logics that govern the interactions between chromatin dynamics, transcripts, proteins, and metabolites. The delineation of “metabolo-epitranscriptional rate equations” refers to mathematical representations of how the concentrations of interacting cellular methylome, gene transcripts, proteins, and metabolites in an MGRN evolve over time in a cell. A “metabolo-epitranscriptional attractor” describes the equilibrium solutions of the metabolo-epitranscriptional rate equations that represent observable cellular states and can be visualized as wells, or depressions, in a metabolo-epigenetic landscape. A “metabolo-epitranscriptional basin of attraction” includes the set of initial conditions of the metabolo-epitranscriptional rate equations that describe how a particular cell moves to particular metabolo-epitranscriptional attractors. It therefore represents a fate-primed subset of a given population, i.e., a particular cellular state.

The concepts of attractors and the associated landscape pictures provide a very useful conceptual framework in considering the metabolo-epigenetic nature of the non-CSC and CSC substates. Although it might be argued that the power of a dynamic perspective of cancer metabostemness using Waddingtonian metabolo-epigenetic landscapes does not extend beyond intuition, we should acknowledge that, in some examples, it has been possible to mathematically describe the relevant attractors and to make predictions about the paths that differentiating cells follow from the undifferentiated, undetermined stem cell state, and vice versa (106, 115, 159). Moreover, the importance of pursuing mathematical models for stemness and differentiation issues should be emphasized when considering that a CSC cellular state should represent a “metabolo-epitranscriptional attractor” in which the maintenance of stemness might not only involve an active process of maintaining this “ground-state,” but also prevent cells from leaving it (160). In this scenario, where CSC cellular states are expected to have an innate program for self-replication that might not require extrinsic instruction, which may certainly account for their latent tumorigenicity, the development of the methods and procedures to mathematically model MGRNs involved in determining self-renewal and pluripotency will not only provide new insights into switch and reprograming properties in cancer tissues, but will also reveal specific, metabolically driven fluctuating behaviors; in particular, the delineation of minimal metabolo-epitranscriptional requirements for cancer self-renewal can pinpoint missing metabolic components and interactions from CSC functionality that can be confirmed in the laboratory, thus generating an iterative procedure in which the occurrence of “metabostemness factors” and “metabolic rules of attraction” is modeled.

**Figure 4:**
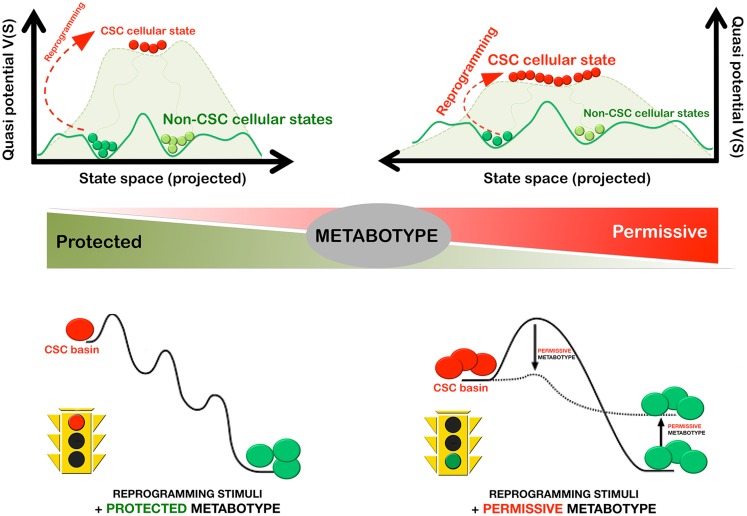
**Metabostemness: metabolic regulation of the efficient and rapid reprograming-to-cancer stemness in cancer tissues**. The cellular metabotype can significantly alter the efficiency and speed of CSC reprograming in cancer tissues by lowering the barriers of the epigenetic landscape and by increasing the size of the basin of attraction of CSC cellular states. In this scenario, even modest changes in the protected versus permissive nature of the cellular metabotype are expected to produce a considerable change on the kinetic efficiency of the reprograming process.

In a first attempt to provide a defined platform for the precise description and dissection of the CSC state in minimal metabolo-epitranscriptional terms, we recently developed the methods and procedures to mathematically model the MGRNs that might be involved in determining pluripotency and self-renewal in mammary epithelial cells; similarly to previous studies, our model formulation considered that the establishment of a balance between Oct4 and Sox2 – the core regulators of pluripotency – and/or counteracting lineage specifiers facilitate reprograming to stemness (98). Our model formulation, however, introduced for the first time ever the epigenetic regulation of the lineage-specific genes, which is an essential element that allows the effects of metabolic regulation in the nuclear reprograming process to be taken into account (Menendez et al., unpublished observations). In particular, we added nucleosome modification by HDM as an essential reprograming element regulated by oncometabolites such as 2-HG. When studying the impact of 2-HG-induced reduction of HDM activity on both the epigenetic landscape of pluripotency and the reprograming kinetics (Figure 4), the following was predicted:

(a) a metabolically driven small reduction of HDM activity should be sufficient to notably lower the barriers of the epigenetic landscape, thus allowing differentiated cells to more easily enter into stem cell macrostates;
(b) the reduction in average reprograming time should vary exponentially with the metabolically imposed reduction of HDM activity, suggesting that even modest metabolic decreases of histone demethylation would produce a considerable increase in reprograming efficiency; and
(c) the metabolic-induced reduction of HDM activity should transfer a portion of the basin of attraction of the differentiated to the pluripotent state thus increasing the size of the basin of attraction of the macrostate occupied by stem cells.

Second, the creation of non-transformed mammary epithelial cells with clinically relevant heterozygous knock-in of the 2-HG-producing IDH1 R132H mutation allowed us to generate proof-of-concept data supporting the actual reprograming activity of oncometabolites predicted in our mathematical model. The experimental validation of functional predictions confirmed the following:

(a) the endogenous accumulation of oncometabolites such as 2-HG is likewise sufficient to significantly enhance and accelerate the efficient generation of induced CSC-like-cells when using the four transcription factors Oct4, Sox2, Klf4, and c-Myc;
(b) even transient exposure to oncometabolites can operate as a microenvironment-driven dedifferentiation event that facilitate “pathological reprograming” because several hours of exposure with a cell-permeable form of 2-HG can fully recapitulate the promoting effects of IDH1 mutations on switching differentiated mammary epithelial cells into induced CSC-like cells; and, more importantly
(c) 2-HG can substitute for Klf4 and c-Myc in reprograming-factor combinations leading to the enhancement and acceleration of reprograming non-CSC into induced CSC-like cells when solely using Oct4 and Sox2.

By impacting the epigenetic remodeling that facilitates the transition between non-CSC and CSC functional states, oncometabolites such as 2-HG likewise appear to operate as bona fide “metabostemness reprograming factors” that determine the cancer tissue proclivity of generating a given number of CSCs in a given time. Because we employed a non-tumorigenic, non-transformed genetic background that, upon introduction of defined reprograming factors, has been found to generate tumorigenic cells with CSC properties (161), our findings demonstrating how small-molecule components of metabolism such as 2-HG efficiently drive significant enhancement and acceleration of CSC reprograming strongly support the notion that metabostemness factors could poise cells with chromatin states competent for rapid dedifferentiation, creating a persistent pre-neoplastic state suitably primed for later transcriptional alterations to finish the reprograming of non-CSC into CSC cellular states.

Given that metabolism represents a junction system receiving cumulative signals from upstream (genome, transcriptome, and proteome) and downstream (microenvironment) systems, pre-malignant, and cancer cells can adapt, resist, or react to multi-omic effects through different types of metabolism and, therefore, based on differential regulation, synthesis, and availability of cellular metabolites. Indeed, all the – omic characteristics that drive cancer plasticity concurrently provide to metabolism a flexibility that can be described by means of, at least, four variability criteria, i.e., the occurrence of certain “elite” metabolites, their concentration levels, their relative levels or ratios between metabolites, and metabolic profiles. The occurrence of particular metabolites should be viewed as a qualitative criterion concerning a strong metabolomic parameter specifically stimulated by particular cancer cell states or that locks cancer cells in a given state by merely being present (e.g., oncometabolites). Although apparently simplistic, this binary aspect of associating unique metabolomic parameters with radically different non-CSC versus CSC cellular “species” is strongly supported by the abovementioned findings with the oncometabolite 2-HG. In this scenario, metabotypes based on the presence–absence of some particular metabolites can have high taxonomic value at specifically differentiating non-CSC versus CSC cellular states in cancer tissues. The increase in the concentration levels of some metabolites could also operate as intrinsic factors governing the proclivity of non-CSC-to-CSC transitions. For instance, the upregulation of the middle glycolysis intermediate fructose-1,6-bisphosphate upon epigenetic silencing of the gluconeogenic enzyme fructose-1,6-biphosphatase is differentially employed by CSCs as a mechanism of glucose flux maintenance and the lowering of reactive oxygen species (ROS) via glycolysis and other associated biosynthetic pathways (127). Forthcoming studies should elucidate whether metabolic ratios between the concentration levels of structurally close metabolites can also provide biochemical functional discrimination between non-CSC and CSC cellular states in heterogeneous cancer populations. In the same regard, should CSC cellular states possess specific changes in the capacities and kinetics of certain metabolic modes, the discovery of unique, CSC-associated metabolic flux imprints will remain a challenge for the field (see below).

### Tissue-dependent cancer metabolic programs: The “metabolic memory” of reprogramed CSC

A dynamic perspective of the metabostemness trait using Waddingtonian metabolo-epitranscriptional landscapes can explain why we are accumulating evidence that tumor cells’ metabolism, often considered a single entity differing from normal cell metabolism, rather exhibits a wide diversity of metabolic phenotypes (162). The heterogeneous expression of metabolic genes is observed across tissue types and, therefore, the metabotypic expression pattern appears to reflect cancer cells’ propensity to adapt the pre-existing metabolic network to successfully support the cancer tissue’s altered needs. Where it has been studied, the metabotype of tumors is a function of both the genetic lesions driving tumorigenesis and the tissue from which the cancer arose (163). Although it has been suggested that this heterogenous metabolic pattern across tissue types can reflect cancer cells’ propensity to adapt the pre-existing metabolic network to support the neoplastic tissue’s altered metabolic needs (162), the metabostemness trait can provide a radically different view of the occurrence of tissue-dependent cancer metabolic phenotypes.

A shift in the balance between mitochondrial OXPHOS and glycolysis that reconfigures the cellular anabolic requirements (i.e., high glycolytic carbon flux and the increased decoupling from ATP production in mitochondria) precedes the appropriate acquisition of stemness traits in iPSCs (10–30, 105). Similar to well-recognized genetic and epigenetic factors, bioenergetics reprograming crucially operates as an enabling regulator of cellular reprograming to stemness because the self-renewal and pluripotency attributes cannot be efficiently acquired in the presence of an inadequate bioenergetic metabotype. Thus, the efficiency of reprograming is higher the closer the glycolytic/OXPHOS energy metabolism profiles of the starting somatic cells are to the pattern observed in ESCs. Moreover, bioenergetics resetting has an early, active role during reprograming because manipulations that inhibit glycolysis reduce, whereas augmenting glycolysis enhances reprograming efficiency, respectively. Therefore, only when a crucial, very early step of engagement to the stemness’ bioenergetics metabotype is correctly initiated can transcriptional regulators of self-renewal and pluripotency then induce additional endogenous factors to acquire a bona fide stem cell cellular state while maintaining the reprograming cells’ bioenergetics’ competence. Accordingly, induced pluripotency can be achieved with a combination of only one stemness transcription factor and small molecules able to facilitate the metabolic transition from mitochondrial OXPHOS to glycolysis. Moreover, there is a progressive resetting of metabolite levels that parallels the progressive changes seen in global epigenetic modifications and gene expression with late passage iPSCs significantly closer to the metabolo-epigenetic profiles of ESCs (19, 164).

The abovementioned findings strongly suggest that, similar to the “epigenetic memory,” a “metabolic memory” also exists that can be partially retained through the reprograming process and might significantly influence the functioning and differentiation potential of iPSCs from cells of different tissues. Although little is known about the bioenergetics resetting of CSCs, it appears that, similarly to iPSCs, a direct link might exist between the occurrence of a metabolic switch from OXPHOS to aerobic glycolysis and the occurrence and maintenance of CSC cellular states. Compared to their more differentiated progeny, the acquisition of a stem cell cellular state might necessarily promote changes in the bioenergetics metabotype because CSCs appear to preferentially perform glycolysis over OXPHOS, at least in some cancer types. Accordingly, recent studies have confirmed that breast CSCs shift from mitochondrial OXPHOS toward fermentative glycolysis and are sensitive to treatment with 2-deoxyglucose, a well-known inhibitor of glycolysis (165). A direct link between glucose metabolism and cancer stem/initiating cells has similarly been established in glioblastoma cancer tissues (166). Once again, by following the numerous parallels between the processes of reprograming differentiated cells-to-iPSCs and differentiated cells-to-CSC, it seems reasonable to suggest that certain bioenergetics features such as the Warburg effect can no longer be viewed as the single metabolic entity shared by all the cancer tissues, but rather as the archetypical aspect of the undifferentiated state owned by CSCs. As recently pointed out by Pacini and Borziani (167), the specific metabolic phenotype known as the Warburg effect might not be considered a metabolic signature that is acquired during the oncogenesis process; conversely, the Warburg effect might represent an aberrant expression of a metabolic layout that is distinctive from the undifferentiated state owned by CSCs. Pacini and Borziani (167) further consider that there is a gradual and irreversible establishment of an undifferentiated state, with a gradual or complete loss of OXPHOS, rather than respiration in itself, which is often present in neoplasms, as originally described by Otto Warburg almost a century ago. Thus, these authors view the permanent shift toward a Warburgian energetic and metabolic state as an essential contributory cause of cancer, as it might represent the central link between genetic/epigenetic instability and the CSC theory considering that a characteristic and essential feature of each neoplasm is the lack of differentiation.

Our metabostemness proposal provides an alternative explanation to the apparently contradictory fact that certain bioenergetic metabotypes such as the OXPHOS-to-glycolysis bioenergetics resetting can be commonly adopted by CSC cellular states from different cancer tissues, whereas, the metabolic networks of individual tumors more closely resemble those of the normal tissue from which the tumor arose than other tumors that develop in different organ sites (168). From a Waddingtonian perspective, establishing a CSC cellular state is an “uphill battle” against the global slope in the landscape, which accounts for the arrow of time of cancer development. Certain metabolic events (e.g., the Warburg effect) might lower the barriers of the epigenetic landscape to provide “smoothly ascending slopes” while concomitantly enlarging the CSC attractor basin and thus promoting the ground-state character of the CSC cell state that is self-maintaining (Figure 4). However, the Warburgian metabolic state of CSC will be nevertheless globally situated at a “high altitude,” which affords the state a strong urge to “differentiate away” and populate all other cancer states attractors situated at a lower “altitude”; upon certain perturbations the metabolo-network state will “flow down” the valleys to the much lower attractors of differentiated cancer cells, which, because of their “metabolic memory” will convey their tissue-specific metabolic patterns without needing any “instructive” signal.

## Cancer Metabostemness: The Challenges Ahead

### Description of the CSC-metabolic phenome: The metabolic importance of the host

Could we determine whether the cellular states of CSCs specifically engage structural or functional changes in metabolic enzymes to produce either specific or differentially enriched CSC-metabolites? We should acknowledge that there are currently no studies on the intracellular metabolic fluxes of CSCs. The elucidation of CSCs’ shift between OXPHOS and aerobic glycolysis based solely on transcriptomic data would imply there are no specific changes in CSC metabolism, as most metabolic changes are not accompanied by significant changes in the transcript levels of metabolic genes. The inclusion of proteomics data would shed further light on CSC-associated metabolism; for example, increased protein expression throughout the glycolytic pathway could suggest that this pathway’s activity is increased in CSC cellular states. However, proteomics data will not be conclusive regarding changes in the activity of certain metabolic pathways. Indeed, detailed knowledge of cell physiology, in particular at the level of cellular metabolism, is entirely lacking for CSCs due to the difficulty of measuring the *in vivo* metabolic fluxes of mammalian cells. A better understanding of these fluxes will be critical in order to exploit the metabostemness trait to eliminate tumor tissues in patients, especially when considering that the tumor cell microenvironment can profoundly affect metabolism and influence how nutrients are metabolized (169, 170). Moreover, tumor tissues are composed of a heterogeneous mixture of cancer cells and normal cells, and symbiotic metabolic relationships have been described in both normal and malignant-tissues contexts (171–175). Because whole body metabolic regulation can also affect tumor tissue metabolism (176), incorporating the complex interplay between genetics, microenvironment, tissue heterogeneity, and whole body metabolism at the CSC-metabolic level remains an enormous challenge for the field. In this regard, we recently reasoned that certain extracellular nutrients might be the major determinant of the maintenance and/or expansion of the metabolic states characteristic of CSC cellular states. Using a standardized high-throughput metabolic phenotyping platform [i.e., the Phenotype MicroArrays for Mammalian Cells (PMM) technology] based on a single-assay metabolic profile of several hundred nutrient sources, we performed a comprehensive evaluation of the phenotypic variations among non-CSC and CSC-like isogenic cells as a starting point for uncovering CSC cells’ microecological nutritional niches (5). Our study revealed that the acquisition of stemness traits by breast epithelial cells is sufficient to cell-autonomously enable the vectorial transfer of energy-rich nutrients from the extracellular microenvironment to energy-producing catabolic pathways in the CSC-like cells. We presented the first evidence for the existence of a cell-autonomous “reverse Warburg effect” where CSC reprograming appears to pre-activate energy-producing pathways that can efficiently use bona fide ketone bodies (e.g., β-hydroxy-butyric acid) and other high-energy metabolites such as lactate and pyruvate from the extracellular milieu to feed mitochondrial energy production, especially upon starvation. Because cancer cells’ utilization of the high-energy metabolites pyruvate, lactate, and ketones has been shown to increase the transcriptional expression of gene profiles normally associated with stemness, including genes commonly upregulated in ESCs (177, 178), our results strongly suggest that global nutrient utilization analyses should be viewed as crucial complements of the metabolo-phenotypic characterization of CSC cellular states.

**Figure 5:**
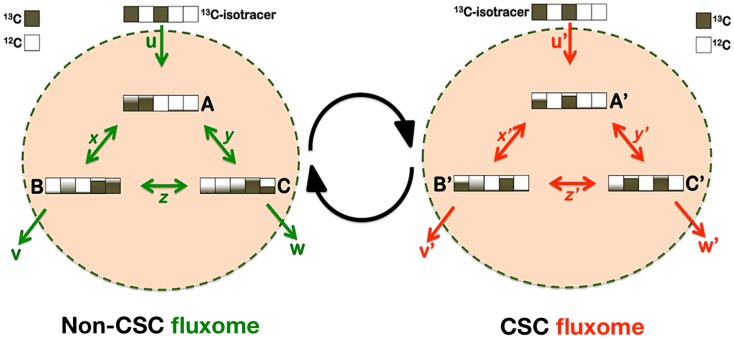
**Metabostemness: a fluxomic perspective**. CSC cellular states should include changes in the capacities (enzyme abundance) and kinetics (enzyme activity) of certain metabolic nodes that might then generate CSC-associated metabolites or metabolomic flux imprints. Metabolic flux analysis (MFA) has become a standard tool to probe cellular metabolism and elucidate *in vivo* metabolic fluxes, which are estimated from isotopic labeling measurements combined with extracellular uptake and excretion rates. Because ^13^C-MFA has been shown to provide an inherently more realistic representation of *in vivo* cellular metabolism, the application of the isotopic ^13^C-MFA methodology might allow obtaining an integrated picture of the metabolic fluxes specifically or differentially occurring in CSC cellular states. In brief, a ^13^C-labeled substrate can be incorporated into the carbon backbone of a wide range of CSC-metabolites and the CSC metabolome, either through exchange or synthesis. The dynamic distribution of labeled carbon traversing along CSC-metabolic pathways is expected to generate a characteristic imprint of labeling patterns whose mass signature will be observed by MS. All fluxes can be determined on an absolute scale because the physiological fluxes in and out of non-CSC and CSC cells will be available under experimental conditions. Mass isotopomers can be analyzed at different times to follow the label incorporation immediately after incubating cells with a labeled substrate. To translate the time-series labeling data into metabolic fluxes, MS-related mathematical models could combine the balances of the total metabolite pools and of individual isotopomers to obtain complete information about the transitions of the labeled carbons within metabolites. The model inputs will be the isotopomer ^13^C time-courses and some metabolite pools determined experimentally, in addition to the consumption or production rates of metabolites measured in cell supernatants. Because the isotopic transient ^13^C-MFA methodology does not rely on uncertain cofactor balances it allows the estimation of fluxes through both parallel and cyclic pathways as well as through bidirectional reactions.

But how should a definitive elucidation of the metabolo-phenomic maps in CSC be approached? Metabolic flux analysis (fluxomics) is known to represent the physiological counterpart of its sibling’s transcriptomics, proteomics, and metabolomics. Fluxomics integrates *in vivo* measurements of metabolic fluxes with stoichiometric network models to allow the determination of absolute flux through large networks of the central carbon metabolism. Thus, fluxomics, by comprising all metabolic conversion rates in a cell, is being increasingly used in fundamental and applied sciences to unravel metabolic infrastructures and activities of metabolic networks and their regulation (Figure 5). Accordingly, forthcoming studies should afford the systematic determination of “CSC fluxomes” by directly inferring the immeasurable *in vivo* central metabolic reaction rates through rigorous mathematical modeling. By performing metabolomic flux analyses using isotopic tracers and mass spectrometry (MS), it might be possible to determine whether CSC cellular states possess highly specific structural or functional changes in metabolic enzymes and nodes that allow them to produce specific or differentially enriched CSC-associated metabolites. CSC fluxomic experiments are currently underway in our laboratory, which consist of feeding the culture of EMT-induced CSC-like cells and isogenic parental cells with a defined ^13^C-labeled substrate, and measuring, through nuclear magnetic resonance (NMR) or MS, the isotopic enrichment in intracellular metabolites. This information is expected to be “stored” in terms of isotopomers (i.e., each of the possible labeling states in which a particular metabolite can be found). The resultant ^13^C-labeling in the intracellular metabolites is expected to impose important constraints on how the labeled carbon substrate is distributed throughout the metabolic network and, hence, on the identity of the non-CSC- and CSC-associated metabolic fluxes.

### Geroncogenesis and metabostemness

The recently proposed “geroncogenesis” hypothesis states that metabolic changes during aging (i.e., the normal decline in oxidative metabolism and the development of a Warburgian glycolytic metabolism in normal tissues) constitute an early and important “hit” that pushes cells toward complete cellular transformation. Wu et al. (179) propose that “is not simply the time taken to accumulate genomic hits that accounts for the increased rate of cancer with age, but the decline in metabolic homeostasis and gene regulation that occurs normally as we age.” The authors place the sirtuins, a family of NAD^+^-dependent deacetylases (180) that have evolved as coordinators of physiological responses to nutrient intake and energetic demand, as central to this aging-induced dysregulation of mitochondrial metabolism. In the geroncogenic scenario, aging induces a gradual reprograming of metabolism toward a “cancer-like” state. This pre-metabolic transformation certainly correlates well with our current proposal of metabostemness, which calls for the need to broaden our current perception on how metabolism and metabolites exert influence over the transcriptional factors, the chromatin structure, and the epigenetic circuits that establish and maintain the self-renewal and differentiation capacities of CSCs. Certain aging-related metabotypic alterations (e.g., a shift toward a predominantly glycolytic, Warburg-like metabolism) might operate as pivotal molecular events rendering any type of cell of origin susceptible to epigenetic rewiring required for the acquisition of aberrant stemness and, concurrently, of refractoriness not only to apoptosis, but also to differentiation. From a geroncogenic perspective (179), certain “gerometabolites” (43) can operate as bona fide metabostemness reprograming factors that poise cells with chromatin states competent for rapid dedifferentiation while concomitantly setting the idoneous metabolic stage for later genetic alterations to finish the reprograming of non-cancerous into tumor-initiating cells. From a dynamic perspective using Waddingtonian landscapes, metabolic changes during aging could causally control or functionally substitute the epitranscriptional orchestration of the genetic reprograming redirecting normal and non-CSC tumor cells toward less-differentiated CSC cellular states by accelerating the number of “stochastic transitions” (i.e., a case in which the metabolo-epitranscriptional rate equations occurring in a given MGRN cannot specify how a cellular state will move from non-CSC attractors to CSC attractors) and increasing the probability of “deterministic transitions” (i.e., a case in which the metabolo-epitranscriptional rate equations occurring in a given MGNR specify how a cellular state will move from non-CSC attractors to CSC attractors once appropriate initial conditions such as genomic hits are defined). By impacting the epitranscriptional remodeling that facilitates the transition between non-CSC and CSC functional states, the metabostemness trait determines the cancer tissue proclivity of generating a given number of CSCs in a given timeframe, thus providing a functional extension of the geroncogenesis hypothesis at the stem cell level. The gero-metabostemness scenario is consistent with the strong association between cancer prevalence and type 2 diabetes, obesity, and sedentary lifestyle and might be counteracted by exercise, calorie restriction (CR), and CR mimetics that may prevent aging tissues from undergoing the metabolic switch in the first place. Forthcoming studies should mechanistically evaluate how organismal diet, stem cell function, and cancer initiation are interconnected (181) and the implications for cancer prevention using metabolic drugs (e.g., biguanides) or natural dietary products including polyphenolic xenohormetins (8, 182, 183).

### Metabostemness and therapeutics: From metabo-stemotoxic drugs to anti-CSC smart foods

The fact that CSC can be generated *de novo* from more differentiated cells adds a crucial concern to the therapeutic elimination of CSC cellular states: how can we resolve the apparently impossible problem of suppressing the molecular biology of the stemness itself as the sole credible target against CSC? The proposed metabostemness hallmark critically contributes to the gaining and retaining of the stem cell-like fate in pre- and malignant-tissues because, as mentioned above, it operates as the physiological glue that connects all the omic layers with a self-autonomous CSC-metabolic quality, thus establishing a therapeutically targetable metabolic continuum in the reprograming-differentiation model of cancer genesis and progression. The metabostemness hallmark not only dictates the plasticity of the original differentiated cell that is targeted so as to be reprogrammable by early or late genetic and/or epigenetic hits, but, by removing or remodeling the potential barriers between non-CSC and CSC cellular states, it can also operate as a bona fide accelerator of the reprograming process, allowing cells to rapidly progress toward the acquisition of a CSC-like status. Because gains and losses of the metabostemness trait can shift the balance of the non-CSC-to-CSC interconversion in one direction or another, it then follows that only certain cell metabotypes will be compatible with the operational properties exclusively owned by CSC cellular states; conversely, certain metabotypes will impose cellular modes refractory to CSC reprograming. In other words, the molecular logic behind the conversion of non-CSC into CSC can be understood in terms of cellular metabotypes that operate as pathways or roadblocks by facilitating or impeding, respectively, the transcriptional events and signal transduction pathways that lastly coordinate the reprograming paths to CSC cellular states. Since metabolic (like epigenetic) programs, and unlike most genetic actors involve in stemness (e.g., loss-of-function mutations in tumor-suppressors, the activation of non-catalytic transcription factors), can be erased, manipulated, and reinitiated, the inclusion of the metabostemness hallmark as a new-dimensional cancer driver opens an entirely new framework for cancer prevention and treatment based on CSCs’ unique metabolic dependencies. Because small perturbations in a particular metabolic pathway or metabolite might have drastic consequences on the formation, maintenance, and evolution of CSC, an unambiguous elucidation of a putative metabo-stem connection remains mandatory before we could develop a new generation of therapies directed against CSCs’ metabolic dependencies. Accordingly, anti-metabolic reprograming strategies begin to provide a roadmap for the generation of novel “metabo-stemotoxic” therapies that metabolically target CSCs in biologically aggressive tumor types; in this regard, perhaps it is not surprising that the potential applications for biguanides in oncology (184) closely relate to their differential metabolic effects during the cellular transformation and CSC stages (185). A growing number of studies have demonstrated that the biguanide metformin selectively ablates CSCs, as evidenced by the decreased expression of pluripotency-associated genes, CSC-associated surface markers, and other CSC-specific properties including tumor-initiation (83, 84, 166, 185–204).

Moreover, a definitive elucidation of the metabolo-phenomic maps of CSC cells may reveal an unforeseen route to the development of therapies against not only the intrinsic metabolic energy-generating machinery of CSC cells, but also their nutritional niches. The emerging discipline of nutritional genomics or nutrigenomics includes both the study of the effects of diet on an individual’s gene activity and health and the study of how genetic composition affects nutrient metabolism (205–208). Through an understanding of the unique roles of specific nutrients and their possible roles in boosting CSC-like cell phenotypes, it might be possible to customize “smart foods” or systemic “metabolic nichotherapies” tailored to the specific nutritional phenomes possessed by cells with CSC cellular states. Nevertheless, because new techniques such as multi-isotope imaging mass spectrometry (MIMS) permit the high-resolution tracking of heavy isotope-labeled molecules upon utilization by specific types of cells (209, 210), the unique metabolic fluxes generated by the catabolic energy-producing machinery of CSC could be implemented in a novel manner to monitor the spatio-temporal distributions and functionality of CSC cellular states in real-time.

## Corollary

In view of recent groundbreaking studies showing that aging-related decline of certain metabolites can set the metabolic stage for later mutations to drive tumorigenesis (i.e., the geroncogenesis hypothesis) and that the epigenetic fate determination of differentiated cells might be converted to pluripotency by microenvironmental cues, it is time to paradigmatically rethink our perception of the regulatory role of metabolic reprograming in cancer cell-fate decisions. Frustrated by the gene-centric guidelines that usually govern of conventional approaches against CSC, we recently envisioned that the incorporation of robust metabolic phenomics, i.e., the systematic acquisition and objective documentation of cancer metabolic data at the level of CSC cellular states, might revolutionize the advancement of CSC-related cancer precision medicine. Our current proposal of the metabostemness cancer hallmark should forge a new and different path to treating and monitoring cancer through the metabolic phenome of CSC cellular states. Under the assumption that the molecular biology of the stemness transformation itself would be the sole credible target in CSC, a systematic combination of biology, biochemistry, pharmacology, genetics, fluxomics, and mathematical approaches should unambiguously provide the first qualitative and quantitative phenomic representation of the metabolic state of CSC, thus unlocking an almost unexplored field of discovery in cancer research. By generating a robust product engine to interrogate the differential cellular metabolism of CSCs relative to normal and non-CSC tumor cells, we might discover an unforeseen new phenomic hallmark of cancer that we have called cancer metabostemness.

**Figure 6:**
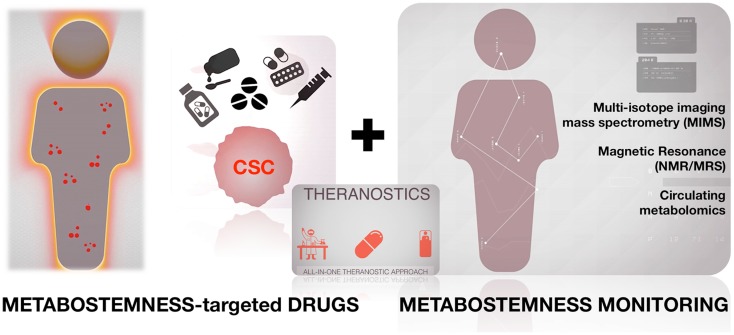
**Metabostemness: a therasnostic perspective**. A qualitative and quantitative representation of the CSC-metabolic state will add a novel dynamic dimension to other well-known cancer hallmarks. Such a description of the CSC-metabolic phenome would make it possible to metabolically create non-permissive (or “hostile”) metabotypes to prevent the occurrence of cellular states with tumor- and metastasis-initiating capacities. In the clinic, the cancer metabostemness attribute will be the basis to rapidly pursue unique therapeutic approaches that target the addiction of CSCs to certain metabolic infrastructures and metabolic fluxes at the cell-intrinsic, microenvironmental, and/or systemic levels. Importantly, the opportunities and challenges for targeting the metabolic infrastructure of CSCs might be rapidly achieved because existing metabolic drugs may be easily repositioned from pre-clinical stages to clinical approaches. For instance, the drugs that would arise from the knowledge-based drug repositioning strategies selected to metabolically suppress the functionality of CSCs could silently operate as “cancer tissue sweepers” of cells capable of initiating and propagating tumors while sparing their normal counterparts. Moreover, differentiating between the specific characteristics of normal tissue SCs and CSCs may be an essential requisite for assessing the best treatment targets for CSCs while minimizing sequelae. The specific, efficient elimination of the malignant teratocarcinoma-initiating cells using the metabolic drug metformin (198) strongly supports the suggestion that the phenomic metabostemness hallmark is a previously unrecognized, indispensable component of the CSC machinery. In addition, the phenomic metabostemness hallmark will allow an “all-in-one” therasnostic approach in cancer diseases, an emerging tool in drug discovery and commercialization that might allow us to take pharmacometabolomics-based precision medicine from the lab to the “point-of-care,” the patient. Should CSC possess specific changes in the capacities and kinetics of certain metabolic nodes, the unique metabolic flux imprints generated by CSC could be revolutionarily implemented to monitor the spatio-temporal distribution and functionality of CSCs in real-time. Coupling metabostemness-based drug testing with the use of non-invasive devices (e.g., MIMS, NMR/MRS, and circulating metabolomics) that can accurately monitor in real-time the spatio-temporal distribution and functionality of CSC could be rapidly implemented to help surgeons, radio-oncologists, and oncologists accelerate and improve metabostemness-based drug discovery and development (note: the illustrations accompanying the figure were created by “Formas Naturales”: http://www.formasnaturales.com/).

Previously, scientists believed that metabolic changes were a mere consequence of aberrant cancer cell growth. We propose that metabolic reprograming of CSC has cancer-causing activity. This proposal suggests an alternative explanation and offers the possibility of delineating a metabolic roadmap for the acquisition and maintenance of CSC cellular states that might not even require pre-existing mutations or rearrangements of well-established “cancer genes.” Moreover, identifying the metabotypic infrastructure of CSCs will add a novel dynamic, phenomic dimension to well-recognized cancer hallmarks. Should CSC possess specific changes in the capacities and kinetics of certain metabolic nodes, the unique metabolic flux imprints generated by CSC could be revolutionarily implemented to monitor CSCs’ spatio-temporal distribution and functionality in real-time. Once in the clinic, this approach can be used to pursue unique and potentially more rapid clinical development pathways by theranostically focusing on cancer populations defined by specific CSC-metabolites or CSC-metabolic imprints. Indeed, we will be able to pharmacologically establish cell metabotypes that are protected against the reprograming events leading to CSC cellular states, thus providing the molecular bases to accelerate and diversify our current therapeutic capacity in new clinical trials designed to metabolically prevent and target CSC cellular states. The metabostemness cancer hallmark generates a shifting oncology theory that should guide a new era of metabolo-epigenetic cancer precision medicine. The tremendous health and social impacts of this paradigmatic shift in the origins of CSC-driven cancer heterogeneity are suited to becoming the platform for new biopharmaceutical strategies based on CSC-metabolic phenomics and dedicated to the research and development of the almost unexplored field of CSC-metabolic theranostics (CSC-metabolic diagnosis + CSC-metabolic treatment; Figure 6) in cancer diseases.

## Conflict of Interest Statement

The authors declare that the research was conducted in the absence of any commercial or financial relationships that could be construed as a potential conflict of interest.

## References

[1] Suvà ML Riggi N Bernstein BE Epigenetic reprogramming in cancer. Science (2013) 339:1567–70 10.1126/science.1230184 23539597 PMC3821556

[2] Menendez JA Joven J Cufí S Corominas-Faja B Oliveras-Ferraros C Cuyàs E The Warburg effect version 2.0: metabolic reprogramming of cancer stem cells. Cell Cycle (2013) 12:1166–79 10.4161/cc.24479 23549172 PMC3674082

[3] Menendez JA Alarcón T Corominas-Faja B Cuyàs E López-Bonet E Martin AG Xenopatients 2.0: reprogramming the epigenetic landscapes of patient-derived cancer genomes. Cell Cycle (2014) 13:358–70 10.4161/cc.27770 24406535 PMC3956532

[4] Campos-Sánchez E Cobaleda C Tumoral reprogramming: plasticity takes a walk on the wild side. Biochim Biophys Acta (2014). 10.1016/j.bbagrm.2014.07.003 25038581

[5] Cuyàs E Corominas-Faja B Menendez JA The nutritional phenome of EMT-induced cancer stem-like cells. Oncotarget (2014) 5:3970–82 24994116 10.18632/oncotarget.2147 PMC4147299

[6] Friedmann-Morvinski D Verma IM Dedifferentiation and reprogramming: origins of cancer stem cells. EMBO Rep (2014) 15:244–53 10.1002/embr.201338254 24531722 PMC3989690

[7] Goding CR Pei D Lu X Cancer: pathological nuclear reprogramming? Nat Rev Cancer (2014) 14:568–73 10.1038/nrc3781 25030952

[8] Menendez JA Joven J Energy metabolism and metabolic sensors in stem cells: the metabostem crossroads of aging and cancer. Adv Exp Med Biol (2014) 824:117–40 10.1007/978-3-319-07320-0_10 25038997

[9] Vicente-Dueñas C Hauer J Ruiz-Roca L Ingenhag D Rodríguez-Meira A Auer F Tumoral stem cell reprogramming as a driver of cancer: theory, biological models, implications in cancer therapy. Semin Cancer Biol (2014). 10.1016/j.semcancer.2014.02.001 24530939

[10] Prigione A Fauler B Lurz R Lehrach H Adjaye J The senescence-related mitochondrial/oxidative stress pathway is repressed in human induced pluripotent stem cells. Stem Cells (2010) 28(4):721–33 10.1002/stem.404 20201066

[11] Chen T Shen L Yu J Wan H Guo A Chen J Rapamycin and other longevity-promoting compounds enhance the generation of mouse induced pluripotent stem cells. Aging Cell (2011) 10:908–11 10.1111/j.1474-9726.2011.00722.x 21615676

[12] Folmes CD Nelson TJ Martinez-Fernandez A Arrell DK Lindor JZ Dzeja PP Somatic oxidative bioenergetics transitions into pluripotency-dependent glycolysis to facilitate nuclear reprogramming. Cell Metab (2011) 14:264–71 10.1016/j.cmet.2011.06.011 21803296 PMC3156138

[13] Folmes CD Nelson TJ Terzic A Energy metabolism in nuclear reprogramming. Biomark Med (2011) 5:715–29 10.2217/bmm.11.87 22103608 PMC3477517

[14] Folmes CD Nelson TJ Dzeja PP Terzic A Energy metabolism plasticity enables stemness programs. Ann N Y Acad Sci (2012) 1254:82–9 10.1111/j.1749-6632.2012.06487.x 22548573 PMC3495059

[15] Folmes CD Dzeja PP Nelson TJ Terzic A Metabolic plasticity in stem cell homeostasis and differentiation. Cell Stem Cell (2012) 11:596–606 10.1016/j.stem.2012.10.002 23122287 PMC3593051

[16] Folmes CD Arrell DK Zlatkovic-Lindor J Martinez-Fernandez A Perez-Terzic C Nelson TJ Metabolome and metaboproteome remodeling in nuclear reprogramming. Cell Cycle (2013) 12:2355–65 10.4161/cc.25509 23839047 PMC3841314

[17] Menendez JA Vellon L Oliveras-Ferraros C Cufí S Vazquez-Martin A mTOR-regulated senescence and autophagy during reprogramming of somatic cells to pluripotency: a roadmap from energy metabolism to stem cell renewal and aging. Cell Cycle (2011) 10:3658–77 10.4161/cc.10.21.18128 22052357

[18] Mahmoudi S Brunet A Aging and reprogramming: a two-way street. Curr Opin Cell Biol (2012) 24:744–56 10.1016/j.ceb.2012.10.004 23146768 PMC3540161

[19] Panopoulos AD Yanes O Ruiz S Kida YS Diep D Tautenhahn R The metabolome of induced pluripotent stem cells reveals metabolic changes occurring in somatic cell reprogramming. Cell Res (2012) 22:168–77 10.1038/cr.2011.177 22064701 PMC3252494

[20] Rafalski VA Mancini E Brunet A Energy metabolism and energy-sensing pathways in mammalian embryonic and adult stem cell fate. J Cell Sci (2012) 125:5597–608 10.1242/jcs.114827 23420198 PMC3575699

[21] Vazquez-Martin A Cufi S Corominas-Faja B Oliveras-Ferraros C Vellon L Menendez JA Mitochondrial fusion by pharmacological manipulation impedes somatic cell reprogramming to pluripotency: new insight into the role of mitophagy in cell stemness. Aging (Albany NY) (2012) 4:393–401 22713507 10.18632/aging.100465 PMC3409676

[22] Vazquez-Martin A Vellon L Quirós PM Cufí S Ruiz de Galarreta E Oliveras-Ferraros C Activation of AMP-activated protein kinase (AMPK) provides a metabolic barrier to reprogramming somatic cells into stem cells. Cell Cycle (2012) 11:974–89 10.4161/cc.11.5.19450 22333578

[23] Vazquez-Martin A Corominas-Faja B Cufi S Vellon L Oliveras-Ferraros C Menendez OJ The mitochondrial H(+)-ATP synthase and the lipogenic switch: new core components of metabolic reprogramming in induced pluripotent stem (iPS) cells. Cell Cycle (2013) 12:207–18 10.4161/cc.23352 23287468 PMC3575450

[24] Corominas-Faja B Cufí S Oliveras-Ferraros C Cuyàs E López-Bonet E Lupu R Nuclear reprogramming of luminal-like breast cancer cells generates Sox2-overexpressing cancer stem-like cellular states harboring transcriptional activation of the mTOR pathway. Cell Cycle (2013) 12:3109–24 10.4161/cc.26173 23974095 PMC3875684

[25] Guan JL Simon AK Prescott M Menendez JA Liu F Wang F Autophagy in stem cells. Autophagy (2013) 9:830–49 10.4161/auto.24132 23486312 PMC3672294

[26] Wang S Xia P Ye B Huang G Liu J Fan Z Transient activation of autophagy via Sox2-mediated suppression of mTOR is an important early step in reprogramming to pluripotency. Cell Stem Cell (2013) 13:617–25 10.1016/j.stem.2013.10.005 24209762

[27] Xu X Duan S Yi F Ocampo A Liu GH Izpisua Belmonte JC Mitochondrial regulation in pluripotent stem cells. Cell Metab (2013) 18:325–32 10.1016/j.cmet.2013.06.005 23850316

[28] Bukowiecki R Adjaye J Prigione A Mitochondrial function in pluripotent stem cells and cellular reprogramming. Gerontology (2014) 60:174–82 10.1159/000355050 24281332

[29] Ito K Suda T Metabolic requirements for the maintenance of self-renewing stem cells. Nat Rev Mol Cell Biol (2014) 15:243–56 10.1038/nrm3772 24651542 PMC4095859

[30] Perales-Clemente E Folmes CD Terzic A Metabolic regulation of redox status in stem cells. Antioxid Redox Signal (2014). 10.1089/ars.2014.6000 24949895 PMC4174422

[31] Ward PS Patel J Wise DR Abdel-Wahab O Bennett BD Coller HA The common feature of leukemia-associated IDH1 and IDH2 mutations is a neomorphic enzyme activity converting alpha-ketoglutarate to 2-hydroxyglutarate. Cancer Cell (2010) 17:225–34 10.1016/j.ccr.2010.01.020 20171147 PMC2849316

[32] Xu W Yang H Liu Y Yang Y Wang P Kim SH Oncometabolite 2-hydroxyglutarate is a competitive inhibitor of α-ketoglutarate-dependent dioxygenases. Cancer Cell (2011) 19:17–30 10.1016/j.ccr.2010.12.014 21251613 PMC3229304

[33] Chowdhury R Yeoh KK Tian YM Hillringhaus L Bagg EA Rose NR The oncometabolite 2-hydroxyglutarate inhibits histone lysine demethylases. EMBO Rep (2011) 12:463–9 10.1038/embor.2011.43 21460794 PMC3090014

[34] Frezza C Pollard PJ Gottlieb E Inborn and acquired metabolic defects in cancer. J Mol Med (Berl) (2011) 89:213–20 10.1007/s00109-011-0728-4 21301796 PMC3043233

[35] Yang M Soga T Pollard PJ Adam J The emerging role of fumarate as an oncometabolite. Front Oncol (2012) 2:85 10.3389/fonc.2012.00085 22866264 PMC3408580

[36] Yang M Soga T Pollard PJ Oncometabolites: linking altered metabolism with cancer. J Clin Invest (2013) 123:3652–8 10.1172/JCI67228 23999438 PMC3754247

[37] Cairns RA Mak TW Oncogenic isocitrate dehydrogenase mutations: mechanisms, models, and clinical opportunities. Cancer Discov (2013) 3:730–41 10.1158/2159-8290.CD-13-0083 23796461

[38] Krell D Mulholland P Frampton AE Krell J Stebbing J Bardella C IDH mutations in tumorigenesis and their potential role as novel therapeutic targets. Future Oncol (2013) 9:1923–35 10.2217/fon.13.143 24295421

[39] Sullivan LB Martinez-Garcia E Nguyen H Mullen AR Dufour E Sudarshan S The proto-oncometabolite fumarate binds glutathione to amplify ROS-dependent signaling. Mol Cell (2013) 51:236–48 10.1016/j.molcel.2013.05.003 23747014 PMC3775267

[40] Adam J Yang M Soga T Pollard PJ Rare insights into cancer biology. Oncogene (2014) 33:2547–56 10.1038/onc.2013.222 23812428

[41] Borger DR Goyal L Yau T Poon RT Ancukiewicz M Deshpande V Circulating oncometabolite 2-hydroxyglutarate is a potential surrogate biomarker in patients with isocitrate dehydrogenase-mutant intrahepatic cholangiocarcinoma. Clin Cancer Res (2014) 20:1884–90 10.1158/1078-0432.CCR-13-2649 24478380 PMC4107454

[42] Gaude E Frezza C Defects in mitochondrial metabolism and cancer. Cancer Metab (2014) 2:10 10.1186/2049-3002-2-10 25057353 PMC4108232

[43] Menendez JA Alarcón T Joven J Gerometabolites: the pseudohypoxic aging side of cancer oncometabolites. Cell Cycle (2014) 13:699–709 10.4161/cc.28079 24526120 PMC3979906

[44] Saha SK Parachoniak CA Ghanta KS Fitamant J Ross KN Najem MS Mutant IDH inhibits HNF-4α to block hepatocyte differentiation and promote biliary cancer. Nature (2014) 513(7516):110–4 10.1038/nature13441 25043045 PMC4499230

[45] Terunuma A Putluri N Mishra P Mathé EA Dorsey TH Yi M MYC-driven accumulation of 2-hydroxyglutarate is associated with breast cancer prognosis. J Clin Invest (2014) 124:398–412 10.1172/JCI71180 24316975 PMC3871244

[46] Hanahan D Weinberg RA Hallmarks of cancer: the next generation. Cell (2011) 144:646–74 10.1016/j.cell.2011.02.013 21376230

[47] Hanahan D Weinberg RA The hallmarks of cancer. Cell (2000) 100:57–70 10.1016/S0092-8674(00)81683-9 10647931

[48] Kroemer G Pouyssegur J Tumor cell metabolism: cancer’s Achilles’ heel. Cancer Cell (2008) 13:472–82 10.1016/j.ccr.2008.05.005 18538731

[49] Menendez JA Lupu R Fatty acid synthase and the lipogenic phenotype in cancer pathogenesis. Nat Rev Cancer (2007) 7:763–77 10.1038/nrc2222 17882277

[50] DeBerardinis RJ Lum JJ Hatzivassiliou G Thompson CB The biology of cancer: metabolic reprogramming fuels cell growth and proliferation. Cell Metab (2008) 7:11–20 10.1016/j.cmet.2007.10.002 18177721

[51] Jones RG Thompson CB Tumor suppressors and cell metabolism: a recipe for cancer growth. Genes Dev (2009) 23:537–48 10.1101/gad.1756509 19270154 PMC2763495

[52] Vander Heiden MG Cantley LC Thompson CB Understanding the Warburg effect: the metabolic requirements of cell proliferation. Science (2009) 324:1029–33 10.1126/science.1160809 19460998 PMC2849637

[53] Locasale JW Serine, glycine and one-carbon units: cancer metabolism in full circle. Nat Rev Cancer (2013) 13:572–83 10.1038/nrc3557 23822983 PMC3806315

[54] Hernandez-Vargas H Sincic N Ouzounova M Herceg Z Epigenetic signatures in stem cells and cancer stem cells. Epigenomics (2009) 1:261–80 10.2217/epi.09.19 22122702

[55] Schoenhals M Kassambara A De Vos J Hose D Moreaux J Klein B Embryonic stem cell markers expression in cancers. Biochem Biophys Res Commun (2009) 383:157–62 10.1016/j.bbrc.2009.02.156 19268426

[56] Maenhaut C Dumont JE Roger PP van Staveren WC Cancer stem cells: a reality, a myth, a fuzzy concept or a misnomer? An analysis. Carcinogenesis (2010) 31:149–58 10.1093/carcin/bgp259 19858069

[57] Floor S van Staveren WC Larsimont D Dumont JE Maenhaut C Cancer cells in epithelial-to-mesenchymal transition and tumor-propagating-cancer stem cells: distinct, overlapping or same populations. Oncogene (2011) 30:4609–21 10.1038/onc.2011.184 21643013

[58] Floor SL Dumont JE Maenhaut C Raspe E Hallmarks of cancer: of all cancer cells, all the time? Trends Mol Med (2012) 18:509–15 10.1016/j.molmed.2012.06.005 22795735

[59] Ben-Porath I Thomson MW Carey VJ Ge R Bell GW Regev A An embryonic stem cell-like gene expression signature in poorly differentiated aggressive human tumors. Nat Genet (2008) 40:499–507 10.1038/ng.127 18443585 PMC2912221

[60] Lengerke C Fehm T Kurth R Neubauer H Scheble V Müller F Expression of the embryonic stem cell marker SOX2 in early-stage breast carcinoma. BMC Cancer (2011) 11:42 10.1186/1471-2407-11-42 21276239 PMC3038979

[61] Liu CG Lu Y Wang BB Zhang YJ Zhang RS Lu Y Clinical implications of stem cell gene Oct-4 expression in breast cancer. Ann Surg (2011) 253:1165–71 10.1097/SLA.0b013e318214c54e 21394007

[62] Liu C Cao X Zhang Y Xu H Zhang R Wu Y Co-expression of Oct-4 and Nestin in human breast cancers. Mol Biol Rep (2012) 39:5875–81 10.1007/s11033-011-1398-6 22207173

[63] Leis O Eguiara A Lopez-Arribillaga E Alberdi MJ Hernandez-Garcia S Elorriaga K Sox2 expression in breast tumours and activation in breast cancer stem cells. Oncogene (2012) 31:1354–65 10.1038/onc.2011.338 21822303

[64] Peng S Maihle NJ Huang Y Pluripotency factors Lin28 and Oct4 identify a sub-population of stem cell-like cells in ovarian cancer. Oncogene (2010) 29:2153–9 10.1038/onc.2009.500 20101213

[65] Yang X Lin X Zhong X Kaur S Li N Liang S Double-negative feedback loop between reprogramming factor LIN28 and microRNA let-7 regulates aldehyde dehydrogenase 1-positive cancer stem cells. Cancer Res (2010) 70:9463–72 10.1158/0008-5472.CAN-10-2388 21045151 PMC3057570

[66] Zhong X Li N Liang S Huang Q Coukos G Zhang L Identification of microRNAs regulating reprogramming factor LIN28 in embryonic stem cells and cancer cells. J Biol Chem (2010) 285:41961–71 10.1074/jbc.M110.169607 20947512 PMC3009922

[67] Beltran AS Rivenbark AG Richardson BT Yuan X Quian H Hunt JP Generation of tumor-initiating cells by exogenous delivery of OCT4 transcription factor. Breast Cancer Res (2011) 13:R94 10.1186/bcr3019 21952072 PMC3262206

[68] Hassiotou F Hepworth AR Beltran AS Mathews MM Stuebe AM Hartmann PE Expression of the pluripotency transcription factor OCT4 in the normal and aberrant mammary gland. Front Oncol (2013) 3:79 10.3389/fonc.2013.00079 23596564 PMC3622876

[69] Madison BB Liu Q Zhong X Hahn CM Lin N Emmett MJ LIN28B promotes growth and tumorigenesis of the intestinal epithelium via Let-7. Genes Dev (2013) 27:2233–45 10.1101/gad.224659.113 24142874 PMC3814644

[70] Boumahdi S Driessens G Lapouge G Rorive S Nassar D Le Mercier M SOX2 controls tumour initiation and cancer stem-cell functions in squamous-cell carcinoma. Nature (2014) 511:246–50 10.1038/nature13305 24909994

[71] Tam WL Ng HH Sox2: masterminding the root of cancer. Cancer Cell (2014) 26:3–5 10.1016/j.ccr.2014.06.024 25026204

[72] Vanner RJ Remke M Gallo M Selvadurai HJ Coutinho F Lee L Quiescent sox2(+) cells drive hierarchical growth and relapse in sonic hedgehog subgroup medulloblastoma. Cancer Cell (2014) 26:33–47 10.1016/j.ccr.2014.05.005 24954133 PMC4441014

[73] Bond JA Oddweig Ness G Rowson J Ivan M White D Wynford-Thomas D Spontaneous de-differentiation correlates with extended lifespan in transformed thyroid epithelial cells: an epigenetic mechanism of tumour progression? Int J Cancer (1996) 67:563–72 10.1002/(SICI)1097-0215(19960807)67:4<563::AID-IJC16>3.0.CO;2-8 8759617

[74] Bonnet D Dick JE Human acute myeloid leukemia is organized as a hierarchy that originates from a primitive hematopoietic cell. Nat Med (1997) 3:730–7 10.1038/nm0797-730 9212098

[75] Reya T Morrison SJ Clarke MF Weissman IL Stem cells, cancer, and cancer stem cells. Nature (2001) 414:105–11 10.1038/35102167 11689955

[76] Al-Hajj M Wicha MS Benito-Hernandez A Morrison SJ Clarke MF Prospective identification of tumorigenic breast cancer cells. Proc Natl Acad Sci U S A (2003) 100:3983–8 10.1073/pnas.0530291100 12629218 PMC153034

[77] Kleinsmith LJ Pierce GB Jr Multipotentiality of single embryonal carcinoma cells. Cancer Res (1964) 24:1544–51 14234000

[78] Pierce GB Speers WC Tumors as caricatures of the process of tissue renewal: prospects for therapy by directing differentiation. Cancer Res (1988) 48:1996–2004 2450643

[79] Passegue E Jamieson CH Ailles LE Weissman IL Normal and leukemic hematopoiesis: are leukemias a stem cell disorder or a reacquisition of stem cell characteristics? Proc Natl Acad Sci U S A (2003) 100:11842–9 10.1073/pnas.2034201100 14504387 PMC304096

[80] Mani SA Guo W Liao MJ Eaton EN Ayyanan A Zhou AY The epithelial-mesenchymal transition generates cells with properties of stem cells. Cell (2008) 133:704–15 10.1016/j.cell.2008.03.027 18485877 PMC2728032

[81] Shackleton M Quintana E Fearon ER Morrison SJ Heterogeneity in cancer: cancer stem cells versus clonal evolution. Cell (2009) 138:822–9 10.1016/j.cell.2009.08.017 19737509

[82] Iliopoulos D Hirsch HA Struhl K An epigenetic switch involving NF-kappaB, Lin28, Let-7 MicroRNA, and IL6 links inflammation to cell transformation. Cell (2009) 139:693–706 10.1016/j.cell.2009.10.014 19878981 PMC2783826

[83] Iliopoulos D Hirsch HA Wang G Struhl K Inducible formation of breast cancer stem cells and their dynamic equilibrium with non-stem cancer cells via IL6 secretion. Proc Natl Acad Sci U S A (2011) 108:1397–402 10.1073/pnas.1018898108 21220315 PMC3029760

[84] Iliopoulos D Hirsch HA Struhl K Metformin decreases the dose of chemotherapy for prolonging tumor remission in mouse xenografts involving multiple cancer cell types. Cancer Res (2011) 71:3196–201 10.1158/0008-5472.CAN-10-3471 21415163 PMC3085572

[85] Gupta PB Fillmore CM Jiang G Shapira SD Tao K Kuperwasser C Stochastic state transitions give rise to phenotypic equilibrium in populations of cancer cells. Cell (2011) 146:633–44 10.1016/j.cell.2011.07.026 21854987

[86] Chaffer CL Brueckmann I Scheel C Kaestli AJ Wiggins PA Rodrigues LO Normal and neoplastic nonstem cells can spontaneously convert to a stem-like state. Proc Natl Acad Sci U S A (2011) 108:7950–5 10.1073/pnas.1102454108 21498687 PMC3093533

[87] Polytarchou C Iliopoulos D Struhl K An integrated transcriptional regulatory circuit that reinforces the breast cancer stem cell state. Proc Natl Acad Sci U S A (2012) 109:14470–5 10.1073/pnas.1212811109 22908280 PMC3437881

[88] Spike BT Wahl GM p53, stem cells, and reprogramming: tumor suppression beyond guarding the genome. Genes Cancer (2011) 2:404–19 10.1177/1947601911410224 21779509 PMC3135646

[89] Magee JA Piskounova E Morrison SJ Cancer stem cells: impact, heterogeneity, and uncertainty. Cancer Cell (2012) 21:283–96 10.1016/j.ccr.2012.03.003 22439924 PMC4504432

[90] Meacham CE Morrison SJ Tumour heterogeneity and cancer cell plasticity. Nature (2013) 501:328–37 10.1038/nature12624 24048065 PMC4521623

[91] Quintana E Shackleton M Foster HR Fullen DR Sabel MS Johnson TM Phenotypic heterogeneity among tumorigenic melanoma cells from patients that is reversible and not hierarchically organized. Cancer Cell (2010) 18:510–23 10.1016/j.ccr.2010.10.012 21075313 PMC3031091

[92] Godar S Ince TA Bell GW Feldser D Donaher JL Bergh J Growth-inhibitory and tumor-suppressive functions of p53 depend on its repression of CD44 expression. Cell (2008) 134:62–73 10.1016/j.cell.2008.06.006 18614011 PMC3222460

[93] Meyer MJ Fleming JM Lin AF Hussnain SA Ginsburg E Vonderhaar BK CD44posCD49fhiCD133/2hi defines xenograft-initiating cells in estrogen receptor-negative breast cancer. Cancer Res (2010) 70:4624–33 10.1158/0008-5472.CAN-09-3619 20484027 PMC4129519

[94] Liu C Kelnar K Liu B Chen X Calhoun-Davis T Li H The microRNA miR-34a inhibits prostate cancer stem cells and metastasis by directly repressing CD44. Nat Med (2011) 17:211–5 10.1038/nm.2284 21240262 PMC3076220

[95] Sharma SV Lee DY Li B Quinlan MP Takahashi F Maheswaran S A chromatin-mediated reversible drug-tolerant state in cancer cell subpopulations. Cell (2010) 141:69–80 10.1016/j.cell.2010.02.027 20371346 PMC2851638

[96] Soda Y Marumoto T Friedmann-Morvinski D Soda M Liu F Michiue H Transdifferentiation of glioblastoma cells into vascular endothelial cells. Proc Natl Acad Sci U S A (2011) 108:4274–80 10.1073/pnas.1016030108 21262804 PMC3060261

[97] Norton L Cancer stem cells, EMT, and seeding: a rose is a rose is a rose? Oncology (Williston Park) (2011) 25:30–2 21361240

[98] Shu J Wu C Wu Y Li Z Shao S Zhao W Induction of pluripotency in mouse somatic cells with lineage specifiers. Cell (2013) 153:963–75 10.1016/j.cell.2013.05.001 23706735 PMC4640445

[99] Yamanaka S Elite and stochastic models for induced pluripotent stem cell generation. Nature (2009) 460:49–52 10.1038/nature08180 19571877

[100] Hanna J Saha K Pando B van Zon J Lengner CJ Creyghton MP Direct cell reprogramming is a stochastic process amenable to acceleration. Nature (2009) 462:595–601 10.1038/nature08592 19898493 PMC2789972

[101] Hanna JH Saha K Jaenisch R Pluripotency and cellular reprogramming: facts, hypotheses, unresolved issues. Cell (2010) 143:508–25 10.1016/j.cell.2010.10.008 21074044 PMC3032267

[102] Zhang Y Li W Laurent T Ding S Small molecules, big roles – the chemical manipulation of stem cell fate and somatic cell reprogramming. J Cell Sci (2012) 125:5609–20 10.1242/jcs.096032 23420199 PMC4067267

[103] Li W Li K Wei W Ding S Chemical approaches to stem cell biology and therapeutics. Cell Stem Cell (2013) 13:270–83 10.1016/j.stem.2013.08.002 24012368 PMC3898630

[104] Rais Y Zviran A Geula S Gafni O Chomsky E Viukov S Deterministic direct reprogramming of somatic cells to pluripotency. Nature (2013) 502:65–70 10.1038/nature12587 24048479

[105] Prigione A Adjaye J Modulation of mitochondrial biogenesis and bioenergetic metabolism upon in vitro and in vivo differentiation of human ES and iPS cells. Int J Dev Biol (2010) 54:1729–41 10.1387/ijdb.103198ap 21305470

[106] Enver T Pera M Peterson C Andrews PW Stem cell states, fates, and the rules of attraction. Cell Stem Cell (2009) 4:387–97 10.1016/j.stem.2009.04.011 19427289

[107] Huang S Reprogramming cell fates: reconciling rarity with robustness. Bioessays (2009) 31:546–60 10.1002/bies.200800189 19319911

[108] MacArthur BD Ma’ayan A Lemischka IR Systems biology of stem cell fate and cellular reprogramming. Nat Rev Mol Cell Biol (2009) 10:672–81 10.1038/nrm2766 19738627 PMC2928569

[109] Visvader JE Cells of origin in cancer. Nature (2011) 469:314–22 10.1038/nature09781 21248838

[110] Visvader JE Lindeman GJ Cancer stem cells: current status and evolving complexities. Cell Stem Cell (2012) 10:717–28 10.1016/j.stem.2012.05.007 22704512

[111] Vermeulen L de Sousa e Melo F Richel DJ Medema JP The developing cancer stem-cell model: clinical challenges and opportunities. Lancet Oncol (2012) 13:e83–9 10.1016/S1470-2045(11)70257-1 22300863

[112] Fessler E Dijkgraaf FE De Sousa E Melo F Medema JP Cancer stem cell dynamics in tumor progression and metastasis: is the microenvironment to blame? Cancer Lett (2013) 341:97–104 10.1016/j.canlet.2012.10.015 23089245

[113] Medema JP Cancer stem cells: the challenges ahead. Nat Cell Biol (2013) 15:338–44 10.1038/ncb2717 23548926

[114] MacArthur BD Lemischka IR Statistical mechanics of pluripotency. Cell (2013) 154:484–9 10.1016/j.cell.2013.07.024 23911316

[115] Morris R Sancho-Martinez I Sharpee TO Izpisua Belmonte JC Mathematical approaches to modeling development and reprogramming. Proc Natl Acad Sci U S A (2014) 111:5076–82 10.1073/pnas.1317150111 24706886 PMC3986203

[116] Lu C Thompson CB Metabolic regulation of epigenetics. Cell Metab (2012) 16:9–17 10.1016/j.cmet.2012.06.001 22768835 PMC3392647

[117] Ward PS Thompson CB Metabolic reprogramming: a cancer hallmark even Warburg did not anticipate. Cancer Cell (2012) 21:297–308 10.1016/j.ccr.2012.02.014 22439925 PMC3311998

[118] Zhang J Nuebel E Daley GQ Koehler CM Teitell MA Metabolic regulation in pluripotent stem cells during reprogramming and self-renewal. Cell Stem Cell (2012) 11:589–95 10.1016/j.stem.2012.10.005 23122286 PMC3492890

[119] Varlakhanova NV Knoepfler PS Acting locally and globally: Myc’s ever-expanding roles on chromatin. Cancer Res (2009) 69:7487–90 10.1158/0008-5472.CAN-08-4832 19773445

[120] Dang CV Le A Gao P MYC-induced cancer cell energy metabolism and therapeutic opportunities. Clin Cancer Res (2009) 15:6479–83 10.1158/1078-0432.CCR-09-0889 19861459 PMC2783410

[121] Dang CV Rethinking the Warburg effect with Myc micromanaging glutamine metabolism. Cancer Res (2010) 70:859–62 10.1158/0008-5472.CAN-09-3556 20086171 PMC2818441

[122] Wahlström T Arsenian Henriksson M Impact of MYC in regulation of tumor cell metabolism. Biochim Biophys Acta (2014). 10.1016/j.bbagrm.2014.07.004 25038584

[123] Goel A Mathupala SP Pedersen PL Glucose metabolism in cancer. Evidence that demethylation events play a role in activating type II hexokinase gene expression. J Biol Chem (2003) 278:15333–40 10.1074/jbc.M300608200 12566445

[124] Liu X Wang X Zhang J Lam EK Shin VY Cheng AS effect revisited: an epigenetic link between glycolysis and gastric carcinogenesis. Oncogene (2010) 29:442–50 10.1038/onc.2009.332 19881551

[125] Wolf A Agnihotri S Munoz D Guha A Developmental profile and regulation of the glycolytic enzyme hexokinase 2 in normal brain and glioblastoma multiforme. Neurobiol Dis (2011) 44:84–91 10.1016/j.nbd.2011.06.007 21726646

[126] Chen M Zhang J Li N Qian Z Zhu M Li Q Promoter hypermethylation mediated downregulation of FBP1 in human hepatocellular carcinoma and colon cancer. PLoS One (2011) 6:e25564 10.1371/journal.pone.0025564 22039417 PMC3198434

[127] Dong C Yuan T Wu Y Wang Y Fan TW Miriyala S Loss of FBP1 by Snail-mediated repression provides metabolic advantages in basal-like breast cancer. Cancer Cell (2013) 23:316–31 10.1016/j.ccr.2013.01.022 23453623 PMC3703516

[128] Liu PP Liao J Tang ZJ Wu WJ Yang J Zeng ZL Metabolic regulation of cancer cell side population by glucose through activation of the Akt pathway. Cell Death Differ (2014) 21:124–35 10.1038/cdd.2013.131 24096870 PMC3857620

[129] Lopez-Serra P Marcilla M Villanueva A Ramos-Fernandez A Palau A Leal L A DERL3-associated defect in the degradation of SLC2A1 mediates the Warburg effect. Nat Commun (2014) 5:3608 10.1038/ncomms4608 24699711 PMC3988805

[130] Hong H Takahashi K Ichisaka T Aoi T Kanagawa O Nakagawa M Suppression of induced pluripotent stem cell generation by the p53-p21 pathway. Nature (2009) 460:1132–5 10.1038/nature08235 19668191 PMC2917235

[131] Kawamura T Suzuki J Wang YV Menendez S Morera LB Raya A Linking the p53 tumour suppressor pathway to somatic cell reprogramming. Nature (2009) 460:1140–4 10.1038/nature08311 19668186 PMC2735889

[132] Li H Collado M Villasante A Strati K Ortega S Cañamero M The Ink4/Arf locus is a barrier for iPS cell reprogramming. Nature (2009) 460:1136–9 10.1038/nature08290 19668188 PMC3578184

[133] Marión RM Strati K Li H Murga M Blanco R Ortega S A p53-mediated DNA damage response limits reprogramming to ensure iPS cell genomic integrity. Nature (2009) 460:1149–53 10.1038/nature08287 19668189 PMC3624089

[134] Utikal J Polo JM Stadtfeld M Maherali N Kulalert W Walsh RM Immortalization eliminates a roadblock during cellular reprogramming into iPS cells. Nature (2009) 460:1145–8 10.1038/nature08285 19668190 PMC3987892

[135] Sarig R Rivlin N Brosh R Bornstein C Kamer I Ezra O Mutant p53 facilitates somatic cell reprogramming and augments the malignant potential of reprogrammed cells. J Exp Med (2010) 207:2127–40 10.1084/jem.20100797 20696700 PMC2947075

[136] Tapia N Schöler HR p53 connects tumorigenesis and reprogramming to pluripotency. J Exp Med (2010) 207:2045–8 10.1084/jem.20101866 20876313 PMC2947071

[137] Bensaad K Tsuruta A Selak MA Vidal MN Nakano K Bartrons R TIGAR, a p53-inducible regulator of glycolysis and apoptosis. Cell (2006) 126:107–20 10.1016/j.cell.2006.05.036 16839880

[138] Kawauchi K Araki K Tobiume K Tanaka N p53 regulates glucose metabolism through an IKK-NF-kappaB pathway and inhibits cell transformation. Nat Cell Biol (2008) 10:611–8 10.1038/ncb1724 18391940

[139] Maddocks OD Vousden KH Metabolic regulation by p53. J Mol Med (Berl) (2011) 89:237–45 10.1007/s00109-011-0735-5 21340684 PMC3043245

[140] Sen N Satija YK Das S PGC-1α, a key modulator of p53, promotes cell survival upon metabolic stress. Mol Cell (2011) 44:621–34 10.1016/j.molcel.2011.08.044 22099309

[141] Sen N Satija YK Das S p53 and metabolism: old player in a new game. Transcription (2012) 3:119–23 10.4161/trns.20094 22771946 PMC3616081

[142] Berkers CR Maddocks OD Cheung EC Mor I Vousden KH Metabolic regulation by p53 family members. Cell Metab (2013) 18:617–33 10.1016/j.cmet.2013.06.019 23954639 PMC3824073

[143] Hitchler MJ Domann FE Metabolic defects provide a spark for the epigenetic switch in cancer. Free Radic Biol Med (2009) 47:115–27 10.1016/j.freeradbiomed.2009.04.010 19362589 PMC2728018

[144] Teperino R Schoonjans K Auwerx J Histone methyl transferases and demethylases; can they link metabolism and transcription? Cell Metab (2010) 12:321–7 10.1016/j.cmet.2010.09.004 20889125 PMC3642811

[145] Wallace DC Fan W Energetics, epigenetics, mitochondrial genetics. Mitochondrion (2010) 10:12–31 10.1016/j.mito.2009.09.006 19796712 PMC3245717

[146] Huang J Sengupta R Espejo AB Lee MG Dorsey JA Richter M p53 is regulated by the lysine demethylase LSD1. Nature (2007) 449(7158):105–8 10.1038/nature06092 17805299

[147] Sauve AA Wolberger C Schramm VL Boeke JD The biochemistry of sirtuins. Annu Rev Biochem (2006) 75:435–65 10.1146/annurev.biochem.74.082803.133500 16756498

[148] Schreiber V Dantzer F Ame JC de Murcia G Poly(ADP-ribose): novel functions for an old molecule. Nat Rev Mol Cell Biol (2006) 7:517–28 10.1038/nrm1963 16829982

[149] Belenky P Bogan KL Brenner C NAD+ metabolism in health and disease. Trends Biochem Sci (2007) 32:12–9 10.1016/j.tibs.2006.11.006 17161604

[150] Quénet D El Ramy R Schreiber V Dantzer F The role of poly(ADP-ribosyl)ation in epigenetic events. Int J Biochem Cell Biol (2009) 41:60–5 10.1016/j.biocel.2008.07.023 18775502

[151] Li X Kazgan N Mammalian sirtuins and energy metabolism. Int J Biol Sci (2011) 7:575–87 10.7150/ijbs.7.575 21614150 PMC3101526

[152] Kim SC Sprung R Chen Y Xu Y Ball H Pei J Substrate and functional diversity of lysine acetylation revealed by a proteomics survey. Mol Cell (2006) 23:607–18 10.1016/j.molcel.2006.06.026 16916647

[153] Wellen KE Hatzivassiliou G Sachdeva UM Bui TV Cross JR Thompson CB ATP-citrate lyase links cellular metabolism to histone acetylation. Science (2009) 324:1076–80 10.1126/science.1164097 19461003 PMC2746744

[154] Zhao S Xu W Jiang W Yu W Lin Y Zhang T Regulation of cellular metabolism by protein lysine acetylation. Science (2010) 327:1000–4 10.1126/science.1179689 20167786 PMC3232675

[155] Choudhary C Kumar C Gnad F Nielsen ML Rehman M Walther TC Lysine acetylation targets protein complexes and co-regulates major cellular functions. Science (2009) 325:834–40 10.1126/science.1175371 19608861

[156] Katada S Imhof A Sassone-Corsi P Connecting threads: epigenetics and metabolism. Cell (2012) 148:24–8 10.1016/j.cell.2012.01.001 22265398

[157] Johnson C Warmoes MO Shen X Locasale JW Epigenetics and cancer metabolism. Cancer Lett (2013). 10.1016/j.canlet.2013.09.043 PMC3984372 24125862

[158] Waddington CH The Strategy of the Genes: A Discussion of Some Aspects of Theoretical Biology. London: Allen & Unwin (1957).

[159] Armond JW Saha K Rana AA Oates CJ Jaenisch R Nicodemi M A stochastic model dissects cell states in biological transition processes. Sci Rep (2014) 4:3692 10.1038/srep03692 24435049 PMC3894565

[160] Ying QL Wray J Nichols J Batlle-Morera L Doble B Woodgett J The ground state of embryonic stem cell self-renewal. Nature (2008) 453:519–23 10.1038/nature06968 18497825 PMC5328678

[161] Nishi M Sakai Y Akutsu H Nagashima Y Quinn G Masui S Induction of cells with cancer stem cell properties from nontumorigenic human mammary epithelial cells by defined reprogramming factors. Oncogene (2014) 33:643–52 10.1038/onc.2012.614 23318426 PMC4697746

[162] Vander Heiden MG Exploiting tumor metabolism: challenges for clinical translation. J Clin Invest (2013) 123:3648–51 10.1172/JCI72391 23999437 PMC3754281

[163] Yuneva MO Fan TW Allen TD Higashi RM Ferraris DV Tsukamoto T The metabolic profile of tumors depends on both the responsible genetic lesion and tissue type. Cell Metab (2012) 15:157–70 10.1016/j.cmet.2011.12.015 22326218 PMC3282107

[164] Chin MH Mason MJ Xie W Volinia S Singer M Peterson C Induced pluripotent stem cells and embryonic stem cells are distinguished by gene expression signatures. Cell Stem Cell (2009) 5:111–23 10.1016/j.stem.2009.06.008 19570518 PMC3448781

[165] Ciavardelli D Rossi C Barcaroli D Volpe S Consalvo A Zucchelli M Breast cancer stem cells rely on fermentative glycolysis and are sensitive to 2-deoxyglucose treatment. Cell Death Dis (2014) 5:e1336 10.1038/cddis.2014.285 25032859 PMC4123079

[166] Sato A Sunayama J Okada M Watanabe E Seino S Shibuya K Glioma-initiating cell elimination by metformin activation of FOXO3 via AMPK. Stem Cells Transl Med (2012) 1:811–24 10.5966/sctm.2012-0058 23197693 PMC3659661

[167] Pacini N Borziani F Cancer stem cell theory and the Warburg effect, two sides of the same coin? Int J Mol Sci (2014) 15:8893–930 10.3390/ijms15058893 24857919 PMC4057766

[168] Hu J Locasale JW Bielas JH O’Sullivan J Sheahan K Cantley LC Heterogeneity of tumor-induced gene expression changes in the human metabolic network. Nat Biotechnol (2013) 31:522–9 10.1038/nbt.2530 23604282 PMC3681899

[169] Yun J Rago C Cheong I Pagliarini R Angenendt P Rajagopalan H Glucose deprivation contributes to the development of KRAS pathway mutations in tumor cells. Science (2009) 325(5947):1555–9 10.1126/science.1174229 19661383 PMC2820374

[170] Metallo CM Gameiro PA Bell EL Mattaini KR Yang J Hiller K Reductive glutamine metabolism by IDH1 mediates lipogenesis under hypoxia. Nature (2011) 481(7381):380–4 10.1038/nature10602 22101433 PMC3710581

[171] Pellerin L Magistretti PJ Glutamate uptake into astrocytes stimulates aerobic glycolysis: a mechanism coupling neuronal activity to glucose utilization. Proc Natl Acad Sci U S A (1994) 91:10625–9 10.1073/pnas.91.22.10625 7938003 PMC45074

[172] Sonveaux P Végran F Schroeder T Wergin MC Verrax J Rabbani ZN Targeting lactate-fueled respiration selectively kills hypoxic tumor cells in mice. J Clin Invest (2008) 118:3930–42 10.1172/JCI36843 19033663 PMC2582933

[173] Kung HN Marks JR Chi JT Glutamine synthetase is a genetic determinant of cell type-specific glutamine independence in breast epithelia. PLoS Genet (2011) 7:e1002229 10.1371/journal.pgen.1002229 21852960 PMC3154963

[174] Salem AF Whitaker-Menezes D Lin Z Martinez-Outschoorn UE Tanowitz HB Al-Zoubi MS Two-compartment tumor metabolism: autophagy in the tumor microenvironment and oxidative mitochondrial metabolism (OXPHOS) in cancer cells. Cell Cycle (2012) 11:2545–56 10.4161/cc.20920 22722266 PMC3404881

[175] Sotgia F Martinez-Outschoorn UE Howell A Pestell RG Pavlides S Lisanti MP Caveolin-1 and cancer metabolism in the tumor microenvironment: markers, models, and mechanisms. Annu Rev Pathol (2012) 7:423–67 10.1146/annurev-pathol-011811-120856 22077552

[176] Pollak M Insulin and insulin-like growth factor signalling in neoplasia. Nat Rev Cancer (2008) 8:915–28 10.1038/nrc2536 19029956

[177] Bonuccelli G Tsirigos A Whitaker-Menezes D Pavlides S Pestell RG Chiavarina B Ketones and lactate “fuel” tumor growth and metastasis: evidence that epithelial cancer cells use oxidative mitochondrial metabolism. Cell Cycle (2010) 9:3506–14 10.4161/cc.9.17.12731 20818174 PMC3047616

[178] Martinez-Outschoorn UE Prisco M Ertel A Tsirigos A Lin Z Pavlides S Ketones and lactate increase cancer cell “stemness,” driving recurrence, metastasis and poor clinical outcome in breast cancer: achieving personalized medicine via Metabolo-Genomics. Cell Cycle (2011) 10:1271–86 10.4161/cc.10.8.15330 21512313 PMC3117136

[179] Wu LE Gomes AP Sinclair DA Geroncogenesis: metabolic changes during aging as a driver of tumorigenesis. Cancer Cell (2014) 25:12–9 10.1016/j.ccr.2013.12.005 24434207 PMC3970212

[180] Feldman JL Dittenhafer-Reed KE Denu JM Sirtuin catalysis and regulation. J Biol Chem (2012) 287:42419–27 10.1074/jbc.R112.378877 23086947 PMC3522242

[181] Mihaylova MM Sabatini DM Yilmaz ÖH Dietary and metabolic control of stem cell function in physiology and cancer. Cell Stem Cell (2014) 14:292–305 10.1016/j.stem.2014.02.008 24607404 PMC3992244

[182] Li Y Wicha MS Schwartz SJ Sun D Implications of cancer stem cell theory for cancer chemoprevention by natural dietary compounds. J Nutr Biochem (2011) 22:799–806 10.1016/j.jnutbio.2010.11.001 21295962 PMC3248810

[183] Menendez JA Joven J Aragonès G Barrajón-Catalán E Beltrán-Debón R Borrás-Linares I Xenohormetic and anti-aging activity of secoiridoid polyphenols present in extra virgin olive oil: a new family of gerosuppressant agents. Cell Cycle (2013) 12:555–78 10.4161/cc.23756 23370395 PMC3594257

[184] Pollak M Potential applications for biguanides in oncology. J Clin Invest (2013) 123:3693–700 10.1172/JCI67232 23999444 PMC3754250

[185] Janzer A German NJ Gonzalez-Herrera KN Asara JM Haigis MC Struhl K Metformin and phenformin deplete tricarboxylic acid cycle and glycolytic intermediates during cell transformation and NTPs in cancer stem cells. Proc Natl Acad Sci U S A (2014) 111:10574–9 10.1073/pnas.1409844111 25002509 PMC4115496

[186] Hirsch HA Iliopoulos D Tsichlis PN Struhl K Metformin selectively targets cancer stem cells, and acts together with chemotherapy to block tumor growth and prolong remission. Cancer Res (2009) 69:7507–11 10.1158/0008-5472.CAN-09-2994 19752085 PMC2756324

[187] Hirsch HA Iliopoulos D Struhl K Metformin inhibits the inflammatory response associated with cellular transformation and cancer stem cell growth. Proc Natl Acad Sci U S A (2013) 110:972–7 10.1073/pnas.1221055110 23277563 PMC3549132

[188] Martin-Castillo B Vazquez-Martin A Oliveras-Ferraros C Menendez JA Metformin and cancer: doses, mechanisms and the dandelion and hormetic phenomena. Cell Cycle (2010) 9:1057–64 10.4161/cc.9.6.10994 20305377

[189] Cufí S Vazquez-Martin A Oliveras-Ferraros C Martin-Castillo B Joven J Menendez JA Metformin against TGFβ-induced epithelial-to-mesenchymal transition (EMT): from cancer stem cells to aging-associated fibrosis. Cell Cycle (2010) 9:4461–8 10.4161/cc.9.22.14048 21088486

[190] Cufi S Corominas-Faja B Vazquez-Martin A Oliveras-Ferraros C Dorca J Bosch-Barrera J Metformin-induced preferential killing of breast cancer initiating CD44+CD24-/low cells is sufficient to overcome primary resistance to trastuzumab in HER2+ human breast cancer xenografts. Oncotarget (2012) 3:395–8 22565037 10.18632/oncotarget.488 PMC3380574

[191] Del Barco S Vazquez-Martin A Cufí S Oliveras-Ferraros C Bosch-Barrera J Joven J Metformin: multi-faceted protection against cancer. Oncotarget (2011) 2:896–917 22203527 10.18632/oncotarget.387 PMC3282095

[192] Jung JW Park SB Lee SJ Seo MS Trosko JE Kang KS Metformin represses self-renewal of the human breast carcinoma stem cells via inhibition of estrogen receptor-mediated OCT4 expression. PLoS One (2011) 6:e28068 10.1371/journal.pone.0028068 22132214 PMC3223228

[193] Oliveras-Ferraros C Cufí S Vazquez-Martin A Torres-Garcia VZ Del Barco S Martin-Castillo B Micro(mi)RNA expression profile of breast cancer epithelial cells treated with the anti-diabetic drug metformin: induction of the tumor suppressor miRNA let-7a and suppression of the TGFβ-induced oncomiR miRNA-181a. Cell Cycle (2011) 10:1144–51 10.4161/cc.10.7.15210 21368581

[194] Vazquez-Martin A Oliveras-Ferraros C Cufí S Martin-Castillo B Menendez JA Metformin and energy metabolism in breast cancer: from insulin physiology to tumour-initiating stem cells. Curr Mol Med (2010) 10:674–91 10.2174/156652410792630625 20712585

[195] Vazquez-Martin A Oliveras-Ferraros C Cufí S Del Barco S Martin-Castillo B Menendez JA Metformin regulates breast cancer stem cell ontogeny by transcriptional regulation of the epithelial-mesenchymal transition (EMT) status. Cell Cycle (2010) 9:3807–14 10.4161/cc.9.18.13131 20890129

[196] Vazquez-Martin A Oliveras-Ferraros C Del Barco S Martin-Castillo B Menendez JA The anti-diabetic drug metformin suppresses self-renewal and proliferation of trastuzumab-resistant tumor-initiating breast cancer stem cells. Breast Cancer Res Treat (2011) 126:355–64 10.1007/s10549-010-0924-x 20458531

[197] Vazquez-Martin A López-Bonetc E Cufí S Oliveras-Ferraros C Del Barco S Martin-Castillo B Repositioning chloroquine and metformin to eliminate cancer stem cell traits in pre-malignant lesions. Drug Resist Updat (2011) 14:212–23 10.1016/j.drup.2011.04.003 21600837 PMC3742095

[198] Vazquez-Martin A Cufi S Lopez-Bonet E Corominas-Faja B Oliveras-Ferraros C Martin-Castillo B Metformin limits the tumourigenicity of iPS cells without affecting their pluripotency. Sci Rep (2012) 2:964 10.1038/srep00964 23236586 PMC3520055

[199] Bednar F Simeone DM Metformin and cancer stem cells: old drug, new targets. Cancer Prev Res (Phila) (2012) 5:351–4 10.1158/1940-6207.CAPR-12-0026 22389436

[200] Song CW Lee H Dings RP Williams B Powers J Santos TD Metformin kills and radiosensitizes cancer cells and preferentially kills cancer stem cells. Sci Rep (2012) 2:362 10.1038/srep00362 22500211 PMC3324825

[201] Lonardo E Cioffi M Sancho P Sanchez-Ripoll Y Trabulo SM Dorado J Metformin targets the metabolic achilles heel of human pancreatic cancer stem cells. PLoS One (2013) 8:e76518 10.1371/journal.pone.0076518 24204632 PMC3799760

[202] Mohammed A Janakiram NB Brewer M Ritchie RL Marya A Lightfoot S Antidiabetic drug metformin prevents progression of pancreatic cancer by targeting in part cancer stem cells and mTOR signaling. Transl Oncol (2013) 6:649–59 10.1593/tlo.13556 24466367 PMC3890699

[203] Würth R Pattarozzi A Gatti M Bajetto A Corsaro A Parodi A Metformin selectively affects human glioblastoma tumor-initiating cell viability: a role for metformin-induced inhibition of Akt. Cell Cycle (2013) 12:145–56 10.4161/cc.23050 23255107 PMC3570504

[204] Zhu P Davis M Blackwelder AJ Bachman N Liu B Edgerton S Metformin selectively targets tumor-initiating cells in ErbB2-overexpressing breast cancer models. Cancer Prev Res (Phila) (2014) 7:199–210 10.1158/1940-6207.CAPR-13-0181 24322659 PMC4497590

[205] Riscuta G Dumitrescu RG Nutrigenomics: implications for breast and colon cancer prevention. Methods Mol Biol (2012) 863:343–58 10.1007/978-1-61779-612-8_22 22359305

[206] Ferguson LR Schlothauer RC The potential role of nutritional genomics tools in validating high health foods for cancer control: broccoli as example. Mol Nutr Food Res (2012) 56:126–46 10.1002/mnfr.201100507 22147677

[207] Levesque JP Winkler IG Rasko JE Nichotherapy for stem cells: there goes the neighborhood. Bioessays (2013) 35:183–90 10.1002/bies.201200111 23129341

[208] Lundstrom K Past, present and future of nutrigenomics and its influence on drug development. Curr Drug Discov Technol (2013) 10:35–46 10.2174/1570163811310010006 22725689

[209] Steinhauser ML Bailey AP Senyo SE Guillermier C Perlstein TS Gould AP Multi-isotope imaging mass spectrometry quantifies stem cell division and metabolism. Nature (2012) 481:516–9 10.1038/nature10734 22246326 PMC3267887

[210] Steinhauser ML Lechene CP Quantitative imaging of subcellular metabolism with stable isotopes and multi-isotope imaging mass spectrometry. Semin Cell Dev Biol (2013) 24:661–7 10.1016/j.semcdb.2013.05.001 23660233 PMC3985169

